# Artificial selection for nonreproductive host killing in a native parasitoid on the invasive pest, *Drosophila suzukii*

**DOI:** 10.1111/eva.13252

**Published:** 2021-06-01

**Authors:** Astrid Kruitwagen, Bregje Wertheim, Leo W. Beukeboom

**Affiliations:** ^1^ Groningen Institute for Evolutionary Life Sciences University of Groningen Groningen The Netherlands

**Keywords:** biological control agents, evolution, genetic improvement, host specificity, host‐parasite interactions, invasive species, parasitoid, pest control, selective breeding, virulence

## Abstract

Establishment and spread of invasive species can be facilitated by lack of natural enemies in the invaded area. Host‐range evolution of natural enemies augments their ability to reduce the impact of the invader and could enhance their value for biological control. We assessed the potential of the *Drosophila* parasitoid, *Leptopilina heterotoma* (Hymenoptera: Figitidae), to exploit the invasive pest *Drosophila suzukii* by focusing on three performance indices: (i) attack rate; (ii) host killing, consisting of killing rate and lethal attack rate (killing efficiency); and (iii) successful offspring development (reproductive success). We found significant intraspecific variation in attack rate and killing rate and lethal attack rate among seven European populations, but offspring generally failed to successfully develop from the *D. suzukii* host. We crossed these European lines to create a genetically variable source population and performed a half‐sib analysis to quantify genetic variation. Using a Bayesian animal model, we found that attack rate and killing rate had a heritability of $h^{2}=0.2$, lethal attack rate $h^{2}=0.4$, and offspring development $h^{2}=0$. We then artificially selected wasps with the highest killing rate of *D. suzukii* for seven generations to test whether host‐killing could be improved. There was a small and inconsistent response to selection in the three selection lines. Realized heritability ($h_{r}^{2}$) after four generations of selection was 0.17 but near zero after seven generations of selection. The genetic response might have been masked by an increased *D. suzukii* fitness resulting from adaptation to laboratory conditions. Our study reveals that native, European, *L. heterotoma* can attack the invasive pest, *D. suzukii* and significantly reduce fly survival and that different steps of the parasitization process need to be considered in the evolution of host‐range. It highlights how evolutionary principles can be applied to optimize performance of native species for biological control.

## 1 INTRODUCTION

The invasion of exotic species can be facilitated by its escape from natural enemies in its native area (Colautti et al., 2004; Keane & Crawley, 2002; Maron & Vilà, 2001). Invasive species can have large detrimental ecological and socio‐economic consequences, including harmful effects on agricultural practices (Paini et al., 2016; Pejchar & Mooney, 2009). Escape from natural enemies can arise due to the inability of native predators and parasite species to find or successfully exploit the invader. For example, native species may not recognize the habitat and/or species‐specific cues associated with the invader (DiTommaso & Losey, 2003; Roy et al., 2011). Also, even if native enemies attack a novel species, they may have no or limited impact on the invader. This can occur when the invasive species has evolved defense strategies to enemies in its area of origin to which natural enemies in the invasive range did not evolve counter defenses, due to a lack of a shared evolutionary history (Desurmont et al., 2011; Gandhi & Herms, 2010). The invader can then act as an evolutionary trap, when the preference of a natural enemy for a prey species is disconnected from its performance (Robertson et al., 2013; Schlaepfer et al., 2002, 2005).

Although the immediate performance of native enemies may be inefficient, when genetic variation exists in their ability to exploit the exotic species, evolution might occur toward higher exploitation efficiency (Carlsson et al., 2009). In particular when the invasive species significantly reduces survival and reproduction ability of the native enemy, it can exert a selection pressure on native enemies' traits resulting in either avoidance or improved detection and exploitation of the invader. Although several examples have been documented indicating that evolutionary change can occur even in relative short time (Ashley et al., 2003; Carroll et al., 2005; Phillips & Shine, 2004; Strauss et al., 2006), the frequency of this host‐range evolution and its consequences for both the invader and native enemy are not clear (Carlsson et al., 2009; Strauss et al., 2006). This is, however, important as knowledge of the evolutionary potential of natural enemies in the invasion area aids pest‐management programs to mitigate biological invasions and to design strategies for augmentative biological control (i.e., the release of additional natural enemies) using already present – native – species (Carroll, 2011; Kruitwagen et al., 2018; Stotz et al., 2016).

A modern strategy in biological pest management is to speed up and direct the evolution of native natural enemies by exploitation of existing intraspecific variation (Kruitwagen et al., 2018; Lommen et al., 2017). Biocontrol agents can be selected and bred with the desired characteristic(s) and then released in the target area (Hoy, 1986; Kruitwagen et al., 2018; Lommen et al., 2017; Wajnberg, 2004). This method has several advantages compared to classical biological control: It mitigates biodiversity risks, reduces nontarget effects (De Clercq et al., 2011), and is not hampered by the Nagoya protocol that impedes the import of exotic natural enemies from the pests' area of origin (Cock et al., 2010; De Clercq et al., 2011; Hajek et al., 2016; van Lenteren, 2012). When novel genetic variants would be introduced through, for example, genetic engineering or introgression of foreign alleles into native species, restrictions may however also apply for selective breeding of native natural enemies. Moreover, it has to be noted that the use of (selected) native biocontrol agents is not always without risks. Selection could for example unintendedly change the agents' host‐range and native biocontrol agents could have nontarget effects or biodiversity risks if they are mass‐released. Quality control and risk assessment are therefore crucial last steps before release (Kruitwagen et al., 2018; Lommen et al., 2017). Yet, selection might not always be feasible and cost‐effective when for example no standing genetic variation is present in the target trait, the target trait is difficult to measure and there is postrelease selection against the traits of interest in nature impairing the establishment of a self‐sustaining population (Kruitwagen et al., 2018; Lommen et al., 2017).

We studied the evolutionary potential of the native parasitoid, *Leptopilina heterotoma* (Hymenoptera: Figitidae), to control the invasive pest species, *Drosophila suzukii* (Diptera: Drosophilidae). This fruit fly invaded and has been spreading through Europe and North America since 2008 (Calabria et al., 2012; Fraimout et al., 2017; Hauser, 2011) and has large economic impact on soft fruit production (De Ros et al., 2015; Farnsworth et al., 2017). Most of the investigated native parasitoid species in the invaded areas have no or limited impact on the invader because *D. suzukii* has a strong immune response against parasitoids (Iacovone et al., 2018; Kacsoh & Schlenke, 2012; Poyet et al., 2013). It also partly inhabits a different niche compared to the *Drosophila* hosts in the invaded area (Atallah et al., 2014; Karageorgi et al., 2017; Keesey et al., 2015), which might impair host finding. The relatively highly virulent larval parasitoid *L. heterotoma* has been found to attack *D. suzukii*, but most investigated populations are not able to complete development on this novel host (Chabert et al., 2012; Knoll et al., 2017; Mazzetto et al., 2016; Poyet et al., 2013). This indicates a mismatch in host selection behavior and reproductive performance, and may impede biological control and host‐range evolution under natural conditions. In the search for new methods of controlling this devastating species in the fruit production industry, it is of interest to assess whether such a mismatch between native parasitoids and the exotic pest species can be amended with evolutionary interventions.

The outcome of parasitization is determined by the progression of stepwise events separated in space and time to pass through different host defenses (Fleury et al., 2009; Gross, 1993; Vinson & Iwantsch, 1980). Parasitization can be divided in host finding, host acceptance, egg laying, and immature development and survival. Although *L. heterotoma* is generally unable to reproduce on *D. suzukii*, it can tackle some of its defense barriers. It is reported to find the host larvae in the field (Miller et al., 2015) and attempt to exploit them by ovipositor insertion (personal observations) and oviposition (Iacovone et al., 2018; Kacsoh & Schlenke, 2012; Poyet et al., 2013; Stacconi et al., 2015). Interestingly, these behaviors can result in nonreproductive host killing (Chabert et al., 2012; Iacovone et al., 2018; Kacsoh & Schlenke, 2012; Mazzetto et al., 2016; Stacconi et al., 2017). This may arise as a result of immune defense costs (encapsulation) of the host (Kraaijeveld et al., 2002; Strand & Pech, 1995), failure of immature parasitoids to fully develop and emerge (“aborted parasitism” sensu Abram et al. (2019)), mechanical damage due to ovipositor insertion (Samson‐Boshuizen et al., 1973), and/or host' exhaustion of counteracting defenses against substances (e.g., venom) injected by the wasp (Asgari & Rivers, 2011; Kohyama & Kimura, 2015; Rizki & Rizki, 1990). Hence, consideration of the stepwise parasitization dynamics may elicit new insights in the formation of host–parasite relationships and their evolutionary potential to evolve to exploit novel hosts (Agrawal & Lively, 2003; Duneau et al., 2011; Elena & Lenski, 2003; Hall et al., 2017; Kaser et al., 2018).

Traits underlying nonreproductive host mortality in parasitoid systems can in part be genetically determined and therefore be subject to evolutionary change (Cavigliasso et al., 2019; Colinet et al., 2013; Henry et al., 2008, 2010; Henter, 1995; Kraaijeveld et al., 2001; Mathe‐Hubert et al., 2019). However, whereas most research focusses on the main outcome of host‐parasitoid interactions, that is, reproductive success, little is known about consequences of the previous steps, such as host‐killing, for population control and evolution of host–parasitoid interactions (Abram et al., 2019). Presence of genetic variation in both reproductive and nonreproductive traits could be exploited for artificial selection to improve biological control efficacy (Kruitwagen et al., 2018). Also, nonreproductive effects might drive adaptive processes (e.g., formation of novel biotic interactions) when traits that determine host‐killing are positively correlated with reproductive success. Alternatively, a negative relationship would constrain adaptation and might endanger population persistence and/or promote selection for host‐range conservation.

In this study, we sought to determine the potential of the native parasitoid *L. heterotoma* to adapt to the novel highly resistant host *D. suzukii*. We first tested and compared European lines on four performance indices (see Section 2.2): attack rate and killing rate, lethal attack rate (killing efficiency, proportion of attacked hosts that are killed), and reproductive success. As we found considerable variation among populations, indicating natural genetic variation, we next crossed these lines to produce a genetically variable strain to further assess the genetic basis underlying these traits for genetic improvement for biocontrol. Key parameters to estimate the amount of genetic variation of a trait and its potential to respond to selection are additive genetic variance and narrow‐sense heritability (Falconer & Mackay, 1996; Lommen et al., 2017). These are defined as the genetic effects that are independent of the genotype in which they occur (thus the main part on which selection acts) and the proportion of the total phenotypic variation due to heritable (i.e., additive) genetic effects, respectively. To estimate additive genetic variance and heritability values, we performed a half‐sib analysis and used a Bayesian “animal model” approach adapted to haplodiploids, to separate additive genetic effects from other sources of variation. This revealed significant genetic variation for host‐killing but not for offspring survival. We next performed seven generations of selection to test the hypothesis that artificial selection can increase nonreproductive killing effects. We also investigated correlated responses of other steps in the parasitization behavior, such as attack rate and reproductive success. Finally, we tested wasp offspring that successfully developed on *D. suzukii* for increased reproductive success on *D. suzukii* in subsequent generations. We consider our results in the context of genetic improvement of this native parasitoid toward the invasive *D. suzukii* and evolutionary ecology of parasitization.

## 2 MATERIAL AND METHODS

### 2.1 Parasitoid and *Drosophila* lines

Seven strains of *L*. *heterotoma* were set up from different European locations: two from the Netherlands (collected from Vosbergen, NL‐Vb, in 2012 and Wageningen, NL‐Wa in 2016), two from Spain (Girona, SP‐Gi in 2016 and Santa Christina d'Aro, SP‐Sa in 2015), and three from France (Saint Etienne sur Chalaronne, FR‐Sa, St Marcel Les Valence, FR‐Sm and Bellegarde, FR‐Be in 2012). Parasitoids were maintained on a relatively low‐resistant *Drosophila melanogaster* host strain (WW) at 25°C, under a light–dark regime of 16:8. These flies were derived from wild flies collected near Leiden, the Netherlands, received in 2009, and kept as mass cultures at 20°C in quarter pint bottles containing 30 ml medium (agar (17 g/L), yeast (26 g/L), sugar (54 g/L), and nipagin (16.7 ml/L)). Parasitoids were tested and selected on *D. suzukii* collected from Westland, the Netherlands in 2016. *Drosophila suzukii* were reared in quarter pint bottles containing 30 ml cornmeal diet (agar (10 g/L), glucose (30 g/L), sucrose (15 g/L), heat‐inactivated yeast (35 g/L), cornmeal (15 g/L), wheat germ (10 g/L), soya flour (10 g/L), molasses (30 g/L), propionic acid (5 ml/L), and Tegosept (2 g/L)).

### 2.2 Standardized parasitization performance test

A standardized test was used for measuring four parasitoid performance indices: attack rate, killing rate, lethal attack rate, and reproductive success. Tested females were at least 5 days old before their performance was measured. To make sure larvae did not die due to “clumsiness” of inexperienced wasps (Samson‐Boshuizen et al., 1973), individual wasps were first given experience with *D. suzukii* larvae for several hours. Next, each individual female was placed in a vial for 4 h with 25 late first‐/second‐instar *D. suzukii* larvae on *Drosophila* medium. Experiments were done at 25°C, and insects had access to a honey droplet on the cotton plug. To measure *D. suzukii* baseline survival in the absence of a parasitoid, at least 10 vials were maintained that were not exposed to parasitoids on each testing day. The number of emerging adult *D. suzukii* flies (*f*) and parasitoid offspring (*p*) were counted to quantify parasitization performances. To account for temporal fluctuations in *D. suzukii* survival over the course of the experiment (1 year), performances were corrected for average fly survival of nonexposed flies (control fly survival, *n*) on the same testing day. The attack rate was the percentage of flies that were parasitized, as estimated from the excess mortality in larvae due to wasp exposure and the number of flies that were attacked but survived (i.e., flies that successfully mounted an immune response (encapsulation)). Encapsulation of wasp eggs by the host was quantified by squashing the flies between two object glasses and inspection under the microscope for presence of a melanized egg. Emerged flies were inspected under the microscope for presence of at least one encapsulated parasitoid egg to quantify the number of hosts that had been parasitized but successfully mounted an immune response (encapsulation) (*e*) and those without capsules (*w*). The percentage of flies that each wasp attacked, corrected for the mortality in nonexposed larvae, was then calculated as:

$$\text{attack rate}=\frac{n‐f‐e}{n}=\frac{n‐w}{n}\cdot 100\%$$

Each wasps' killing rate was calculated as the percentage of flies killed in excess to the mortality in nonexposed flies:

$$\text{killing rate}=\frac{n‐f}{n}\cdot 100\%$$

When the killing rate was negative for an individual (i.e., *f* > *n*), it was set to zero (i.e., *f* was set to *n*). The efficiency at which flies were killed was calculated as the proportion of flies killed from the total number of “attacked” flies:

$$\text{lethal attack rate}=\frac{\text{killing rate}}{\text{attack rate}}=\frac{\left(n‐f\right)/n}{\left(n‐w\right)/n}=\frac{n‐f}{n‐w}$$

The proportion of killed flies that yielded wasp offspring was measured as indication of each individuals' successful parasitism:

$$\text{successful parasitism}=\frac{p}{n‐f}\cdot 100\%$$

### 2.3 Establishment of a genetically diverse line

European populations of *L. heterotoma* were first tested and compared for their ability to parasitize *D. suzukii* following the standardized individual performance test. Next, a genetically diverse laboratory strain was created to estimate heritability of parasitization performances and as starting point for artificial selection on nonreproductive host killing following a reciprocal crossing scheme using the seven European populations. This method ensured equal genetic contribution of all wasp strains and could potentially lead to new allelic combinations. Moreover, it enabled monitoring of potential masked effects of mating preferences, incompatibilities (e.g., due to *Wolbachia* presence) and deleterious effects of homozygotes. Unmated males and females from two different lines were put together (without hosts) for 3–5 days to assure mating. Next, females were placed on second instar *D. melanogaster* larvae to reproduce. No signs of unviability or high male/female biased sex ratio (>70%) were detected in the F2 except for crosses between FR‐Sa and FR‐Be, which were highly male biased. These were therefore repeated to ensure their genetic contribution to the mix population.

### 2.4 Heritability—half‐sib design

Half‐sib families were created by mating one male (sire) with three virgin females (dams), and three offspring of each female were tested for parasitization performances. First, parents (P) were randomly collected from the genetically diverse line by separation of pupae in individual vials prior to emergence of the adult wasps. Next, 1‐ to 3‐day‐old unmated males were allowed to mate with three unrelated 1‐ to 3‐day‐old virgin females by placement in vials with agar (simultaneously). They were provided with honey as food and kept at 25°C for 3 days. To generate a new generation (F1), each female was provided with 20–30 *D. melanogaster* larvae on our standard WW rearing medium to parasitize for 24 h. This setup ensures relatively high‐quality hosts, as prior tests showed high host survival rate (>90%) in the absence of wasps (A. Kruitwagen et al., unpublished data). Three daughters per female were tested for parasitization performances. To reduce common environmental effects when comparing siblings (i.e., similarities within families due to shared environmental experience rather than genetic differences), F1 females were placed on three different host batches each for 24 h. Female F2 offspring were allowed to mate with their brothers, by keeping offspring from each host‐batch in each vial for at least 3 days after emergence. Next, from each host‐batch, one F2 female was randomly selected and tested. Female offspring were tested for the four parasitization performance components. Due to practical restrictions, families were tested in five different blocks. Offspring performances of each block were tested on the same day.

### 2.5 Artificial selection for killing rate

Wasps from the genetically diverse population (P) were randomly divided over six lines: three selection (S) and three control lines (C), each consisting of 100 males and 100 females. Due to the time‐intensive nature of setting up each generation and the artificial selection procedure, this was the largest possible population size in this study system that allowed for imposing a selection regime while limiting influences of inbreeding, genetic drift and rapid depletion of genetic diversity (Fry, 2003; Weber & Diggins, 1990). Control lines were kept to investigate whether any change over time was due to other causes rather than response to selection on killing. The three replicate selection lines were set up to distinguish selection from drift, as consistent changes in the same direction applied across all replicate lines relative to the unselected control lines is unlikely to be due to drift, as drift acts in a random manner. Moreover, we estimated the realized heritability ($h_{r}^{2}$), the response to selection as proportional to the amount of selection applied, to quantify the degree of phenotypic change due to selection (see below) (Falconer & Mackay, 1996; Lynch & Walsh, 1998). Each line was selected for seven successive generations (F1–F7).

Each generation, phenotypic variation was quantified within each replicate line after which the best‐performing individuals were selected and cultured. Killing rate was measured of each individual female following the standardized performance test, and the 50 (out of 100) females with the highest trait value were chosen to contribute to the next generation. Besides selection for killing rate, attack rate and reproductive success were measured to investigate potential correlated response to selection. Due to logistics, individuals from each line were tested over a period of 2–5 days. Selection of 50% of the individuals was chosen to reduce inbreeding, genetic drift and chance of rapid depletion of genetic diversity. In particular, as host killing is a trait potentially controlled by many genes, strong selection of a small proportion could increase the chance of losing beneficial alleles and thus deplete genetic diversity and reduce response to selection.

Parasitization of *D. suzukii* during the performance tests rarely yielded wasp offspring. Therefore, the highest performing mothers were placed individually in vials on the low resistant host, *D. melanogaster*, to generate the next generation. Offspring were collected by taking eight random parasitoid pupae from each of the mothers just before emergence, and these were then divided over two agar bottles (one served as back‐up). This allowed the offspring to mate among each other and thus reduced chances of sib‐mating. Offspring were kept in the agar bottles at 20°C until the performance of 100 randomly chosen females was measured and compared again (hence each line always remained at the constant size of 100 females). The same protocol was followed for the control lines except that each generation 50 random females were chosen to contribute to the next generation. Moreover, as phenotypic evaluations are labor‐intensive, killing performance of control lines could only be tested and compared to the selection lines within generations 5 and 7 of artificial selection.

### 2.6 Repeated selection on reproductive success

During artificial selection on killing rate, parasitoids were occasionally able to successfully reproduce on *D. suzukii*. To test whether offspring that emerged from *D. suzukii* differed in *D. suzukii* exploitation performances and whether this could increase offspring developmental success, a separate selection line was created from *D. suzukii* reproducers (R1) alongside the three artificial selection and control lines, and subjected to selection for reproductive success. Offspring that emerged during each generation of the selective breeding on killing rate were added to the R1 and used as starting material for selection on reproductive success. Females of the R1 were tested following the standardized performance test, and offspring that emerged from *D. suzukii* were selected and used to set up a new selection line (R2). Females of the R2 line were then again tested and offspring that emerged from *D. suzukii* were collected and used to set up a third line, R3. Note that the R1 wasps were bred alongside the selection on killing rate and after each round of selection (P‐F7) on killing rate new genetic material was added to the R1. We therefore decided to test and select the R1 on reproductive success each generation after new parasitoids were added to the line, resulting in a repeated selection process of the same line but each time with the novel added genotypes. The R1 was subjected to eight selection rounds. Similarly, the R2 was bred simultaneously with the R1, and was also repeatedly tested and selected as new genetic material was added to the population from the R1 (in total 8 times selected). To test whether the line selected for repeated reproductive success on *D. suzukii*, R3, differed in killing rate and reproductive success, the R3 line was tested and compared to the base population (P) which was used as starting point of selection and the selection line that best responded to selection on host‐killing (S2), following the individual exploitation performance test.

### 2.7 Statistical analysis

All analyses were done in R (version 3.6.1) (R Core Team, 2020).

#### 2.7.1 Half‐sib analysis

Each parasitization performance trait was analyzed by specifying a two‐column matrix with the number of “success” and “failures,” using the “multinomial2” family argument (Hadfield, 2010). As parasitization performances are also expected to be partly determined by the fitness of the fly hosts, models were also fitted by taking variation in fly survival into account between the five testing days by standardizing parasitization performances with the average fly survival of the controls that were not exposed to wasps. Animal ID was fitted as random effect to estimate the additive genetic variance (de Villemereuil, 2012). Moreover, measurement day and mother ID were taken as random effects to account for similarities between individuals measured on the same day and for influence of the mother. One block was omitted because the average *D. suzukii* survival of the control group was about half of the other four blocks (10.8, vs 21.1, 20.0, 22.0, 20.0), suggesting a low‐quality host batch, which makes estimation of parasitization performances of these wasps unreliable. From the models, we computed the quantitative genetic parameters: additive genetic variance and heritability. The narrow sense heritability $\left(h^{2}\right)$ was estimated by dividing the genetic variance component by the total phenotypic variance (*V_a_*/*V*_total_). We added 1 to the denominator due to the probit link function.

Weakly uninformative priors were chosen. The residual variance (*V_r_*) was fixed to 1 as for binomial‐related families the residual variance is not identifiable (de Villemereuil, 2012). Note that *V_a_* scales with the value of *V_r_*, meaning that heritability estimates are roughly nonsensitive to the actual value to which *V_r_* is fixed (Pierre de Villemereuil, personal communication). For the random effects (which includes *V_a_*), the inverse‐Gamma prior is advised and commonly used (*V* = 1, nu = 0.002 in MCMCglmm) (Hadfield, 2010). However, as this places too much weight on 1 when estimating heritability in binomial traits, following de Villemereuil (2012) and de Villemereuil et al. (2013), we used the Chi‐square distribution with 1 degree of freedom (*V* = 1, nu = 1000, alpha.mu = 0, alpha. *V* = 1 in MCMCglmm). This improves the rate of convergence and shows a relatively close uniform distribution of heritability. Posterior distributions were sampled 910,000 times. Autocorrelation (<0.1) and effective sample size (>1000) were verified to increase confidence in parameter estimates and convergence was tested with the Heidelberg stationary test (de Villemereuil, 2012; Wilson et al., 2010).

#### 2.7.2 Parasitization performances of European populations and selection lines

Fly survival and parasitization performance indices were analyzed with generalized mixed models for binomial data by specifying a two‐column matrix with the number of “successes” and “failures” using the lme4 package (Bates et al., 2014). The response to selection was analyzed by fitting fly survival as dependent variable and treatment (wasp presence/absence), generation (as continuous) and their interaction as fixed factors, and date and line as random factors. Parasitization performances between wasp lines were compared by correcting for day‐to‐day variation in fly survival. To this end, performances were standardized with the average fly survival of the control flies that were not exposed to wasps on the same testing day (c). We tested for overdispersion by comparing the sum of squared Pearson residuals to the residual degrees of freedom following Bolker et al. (http://bbolker.github.io/mixedmodels‐misc/glmmFAQ.html#overdispersion). When overdispersion was detected (*α* = 0.05), observation‐level random effect was added (Harrison, 2015). Significance of main effects was tested by comparing the full model to the model without the fixed effect by ANOVA. Post hoc comparison of means was performed with Tukey test, using the emmeans package (Lenth, 2020).

#### 2.7.3 Realized heritability during artificial selection

The response to selection also provides an estimate of realized heritability ($h_{r}^{2}$) of killing rate, which can be estimated as the slope from the linear regression of the cumulative response to selection over the cumulative selection differential forced to the origin (Falconer & Mackay, 1996). For each replicate line, we calculated the response to selection (R) as the mean offspring value minus the mean of the total parental population, and the selection differential (S) as the mean of the selected parents minus the mean of the total parental population. Phenotypic values were calculated as the percentage of flies killed standardized for the average fly survival of flies not exposed to wasps.

## 3 RESULTS

### 3.1 Population differences in parasitization performance

**FIGURE 1:**
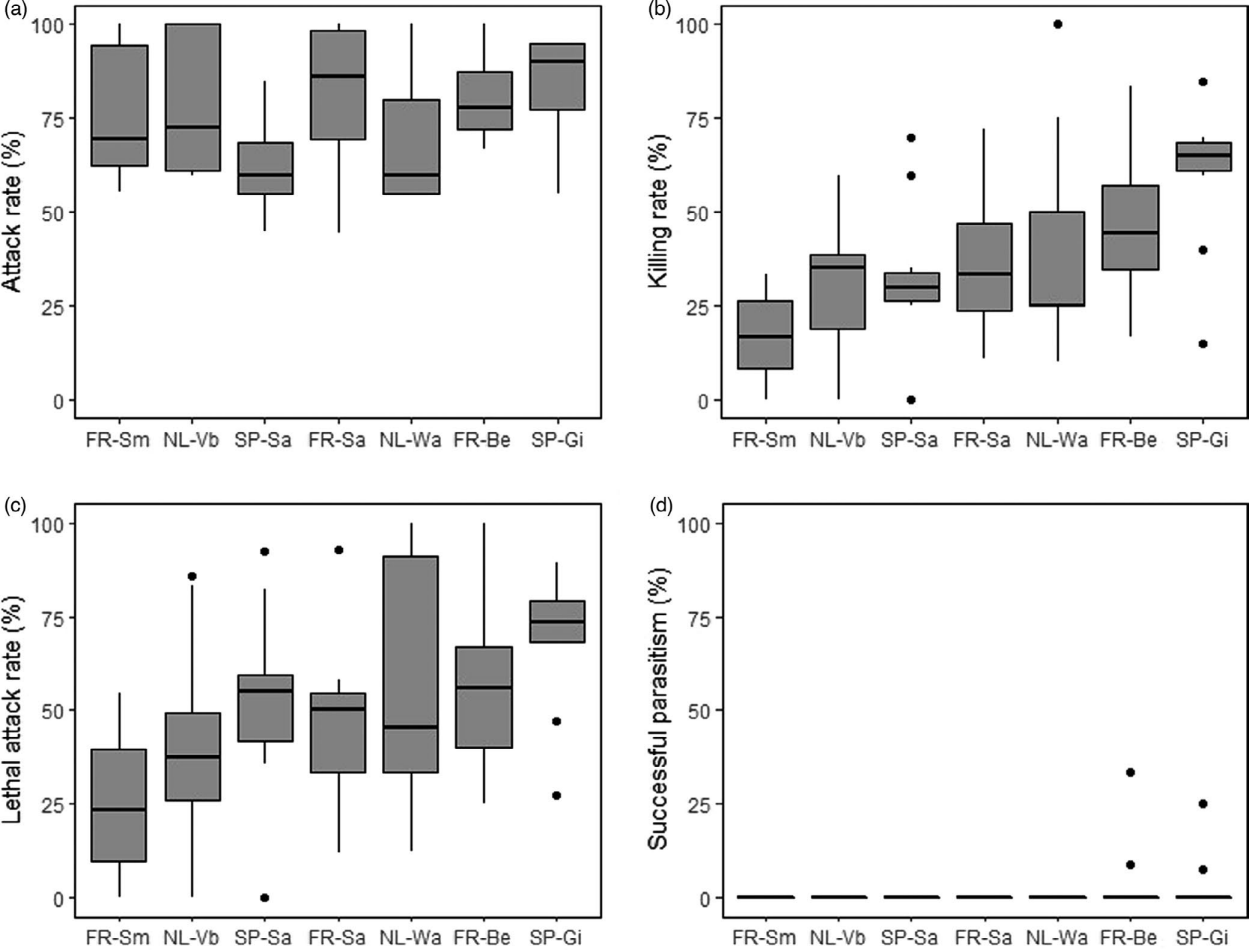
Parasitization performances of European *Leptopilina* *heterotoma* populations attacking *Drosophila* *suzukii*: (a) attack rate, the percentage of killed flies plus flies that survived with encapsulated wasp egg, (b) killing rate, the percentage of flies killed, (c) lethal attack rate, the percentage of flies killed out of the total flies attacked, (d) successful parasitism, the percentage of killed flies that yielded wasp offspring (see Section 2). Boxplots provide data for ten replicates per strain, each replicate containing 30 larvae that were exposed to two parasitoid females. Abbreviations: FR, France; NL, The Netherlands; SP, Spain, for population name abbreviations see Section 2. Horizontal lines represent median, top and bottom are 25th and 75th percentiles and points' outliers

Females of *L. heterotoma* of all seven European populations readily accepted *D. suzukii* hosts for parasitization (Figure 1a). Parasitization by *L. heterotoma* significantly reduced *D. suzukii* survival (GLMM, *β* = −0.97, *χ* ^2^ (1) = 5.77, *p* = 0.016), and killed on average 37.4% ± 2.74 SE of the flies with a range of 0–100%. Populations differed in killing rate (i.e., the percentage of flies killed after adjustment for fly mortality of nonexposed larvae) (GLMM, *χ* ^2^ (6) = 122.38, *p* < 0.01) (Figure 1b): the FR‐Sm line had a significantly lower killing rate compared to NL‐Wag, FR‐Be, and SP‐Gi, and the SP‐GI population showed a higher killing rate than SP‐Sa and NL‐VB (Tukey's post hoc test, *p* < 0.05). The attack rate approached significance (GLMM, *χ* ^2^ (6) = 12.28, *p* = 0.056) (Figure 1a), but the proportion of attacked flies that were killed differed significantly (Figure 1c) (GLMM, *χ* ^2^ (6) = 19.52, *p* = 0.003), and ranged between 0 and 100% with an average of 49.3% ± 3.24 SE. The FR‐Sm population was less efficient in host‐killing compared to FR‐BE, SP‐Gi, and NL‐Wa (Tukey post hoc test, *p* < 0.001). The percentage of killed flies that yielded offspring did not differ (GLMM, *χ* ^2^ (6) = 3.03, *p* = 0.8), and was nearly zero in all populations (Figure 1d). Of the 2070 fly hosts that were exposed to parasitoids, only five yielded offspring (0.24%) (2 by SP‐Gi, and 3 by FR‐Be). In other experiments, parasitoids of FR‐Sa and NL‐Wa also occasionally successfully reproduced on *D. suzukii* (A. Kruitwagen, unpublished data).

### 3.2 Additive genetic variation and heritability of parasitization performances

**FIGURE 2:**
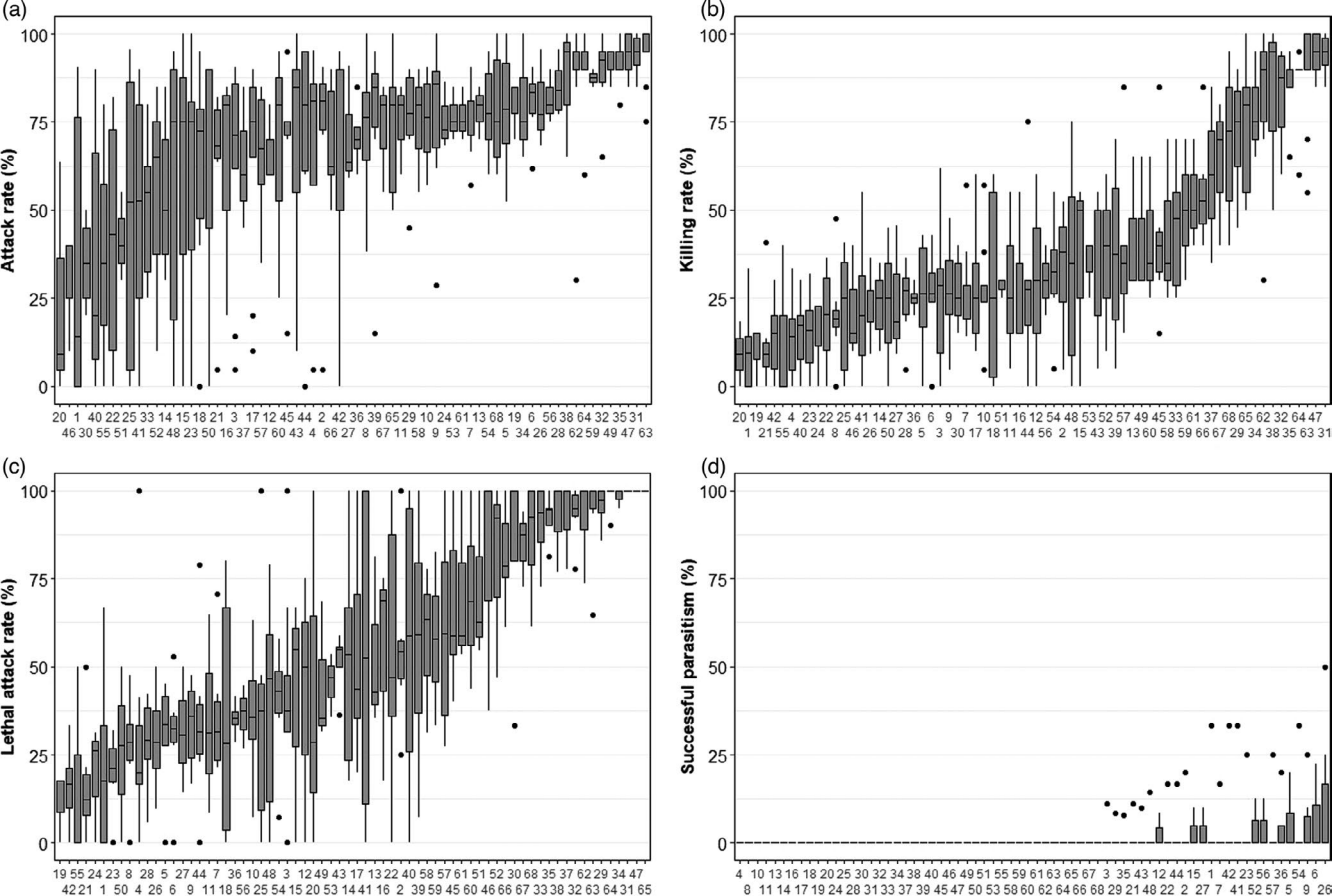
Parasitization performances per half‐sib family (sire), ordered per trait on median values. Performances were standardized for the average fly mortality of nonexposed flies. Horizontal lines represent median, top, and bottom are 25th and 75th percentiles and points are outliers

In total 68 sires, 122 dams and 357 offspring were analyzed. Not each sire mated successfully with the three females provided: 33 sires mated with two dams and 28 with one dam. Offspring of each female were tested for their parasitization performances on *D. suzukii*. Killing rate, attack rate, and lethal attack rate all showed large intraspecific phenotypic variation ranging from 0% to 100% and related individuals exhibited comparable parasitization performance; this was not the case for successful parasitism (Figure 2). Animal models demonstrated significant additive genetic effects underlying attack rate, killing rate and lethal attack rate but not for successful parasitism which had (almost) zero genetic variance (Table 1). Day of measurement (families were tested in four different blocks) also influenced phenotypic variation, indicated by a significant effect of “testing day” (Table 1), but “mother” did not, which includes variance due to common maternal environment. This nongenetic factor thus also contributes to the observed phenotypic variation in parasitization performances. Heritability, that is, the proportion of phenotypic variation due to additive genetic effects, was significantly higher than zero for killing rate, attack rate and lethal attack rate (Table 1). Heritability was about 0.1–0.2 larger when standardized for natural fly survival, indicating that environmental or host effects influenced trait value expression. Attack rate showed low to moderate heritability (0.22, 0.44), depending on whether it was standardized or not, and was larger than the comparable models for host killing rate (0.15, 0.28). Unstandardized lethal attack rate (killing efficiency) showed moderate heritability (0.5), whereas standardized lethal attack rate had the largest heritability of 0.6 and successful parasitism the lowest, that is, close to zero.

**TABLE 1:** Variance components and heritability of parasitization performances of *Leptopilina* *heterotoma* tested on *Drosophila suzukii* hosts from Bayesian animal models

	Variance components				Heritability (*h* ^2^)
	Additive genetic variance (*V_a_*)	Testing day	Mother	Residual variance	
Killing rate	0.413 (0.256–0.688)	0.2434 (0.127–2.617)	0.000367 (7.324E−7–0.1304)	1	0.148 (0.063–0.217)
Std. killing rate	1.077 (0.7406–1.553)	0.3453 (0.0426–3.1867)	0.0009817 (5.653e−7–0.211)	1	0.281 (0.132–0.372)
Attack rate	0.658 (0.287–1.117)	0.0179 (1.234E−7–1.093)	0.0022149 (1.043e−06–0.372)	1	0.223 (0.106–0.363)
Std. attack rate	1.886 (1.091–1.054)	0.00941 (3.108E−5–0.766)	0.001456 (4.203e−7–0.296)	1	0.442 (0.329–0.579)
Lethal attack rate	2.784 (1.819–3.831)	1.0098 (0.171–3.434)	0.0010463 (1.809e−09–0.219)	1	0.484 (0.305–0.583)
Std. lethal attack rate	4.656 (3.362–6.462)	0.6522 (0.251–4.493)	0.0015981 (9.273e−07–0.2629)	1	0.606 (0.378–0.700)
Successful parasitism	0.01052 (2.172e−6–1.364)	0.1963 (5.152e−04–2.668)	0.007828 (1.177e−06–1.263)	1	0.001757 (7.648e−07–0.318)
Std. successful parasitism	0.004270 (2.274e−7–0.989)	0.004372 (1.659e−6–1.524)	0.00885 (1.386e−5–0.987)	1	0.002066 (9.021e−08–0.296)

Note

Models were compared with and without standardization for average *Drosophila* *suzukii* mortality on the same measurement day when not exposed to wasps. Point estimates are posterior mode and ranges are 95% credible interval of the posterior mode, that is, the range of values that the parameter takes with 95% probability. Note that residual variance was set to 1 due to nature of the data (see Section 2).

### 3.3 Artificial selection for killing rate

#### 3.3.1 Response to selection: killing rate

**FIGURE 3:**
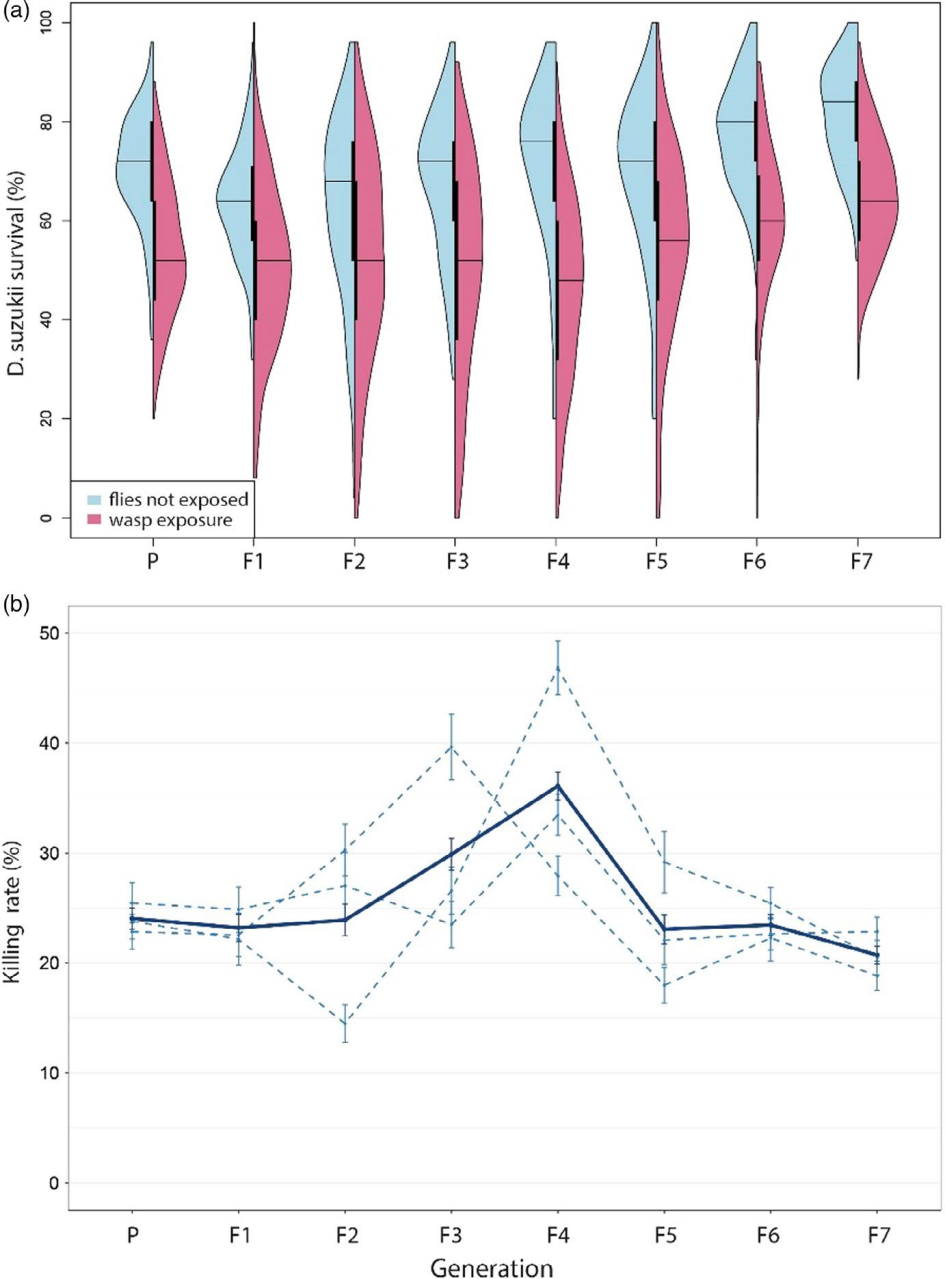
Response to artificial selection of *Leptopilina* *heterotoma* for increased killing rate. Survival of *Drosophila* *suzukii* (a): red violin plots (on the right) depict host survival when exposed to wasps of the three selection lines combined (*n* = 300), and blue violin plots (on the left) depict survival of nonexposed flies (*n* = 112–171). Horizontal lines represent median host survival and inner vertical lines the interquartile range. (b) The difference in mortality between exposed (red) and nonexposed (blue) flies Average killing rate was calculated as the percentage of flies killed, adjusted for mortality in nonexposed fly, of the three selection lines combined (dark blue, strait line), and of each selection line separately (light blue dotted line, *n* = 100). Error bars represent standard deviations of the mean

Fly survival in absence and presence of wasps was variable within and between generations, but overall fly survival was significantly reduced when exposed to parasitoids (Figure 3a) (GLMM, *β* = −0.71, *χ* ^2^ (1) = 122.38, *p* < 0.01). The average *D. suzukii* survival in absence of parasitoids was relatively constant during the first two generations, but increased from the F2 onwards. The average survival rate in the F7 in the absence of wasps was 30% higher compared to the F2. This trend was supported by observed changes in fly rearing quality, that is, increased fecundity and survival. The percentage of flies that parasitoids were able to kill initially ranged from 0 to 69.3% with an average of 24.0% ± 0.96SE (Figure 3b). During the first four generations of selection, the average killing rate increased to 36.1% ± 1.27 SE (ranging from 0 to 100%) and the percentage of individuals that killed >40% flies increased from 17% to 43%. During the last three generations, average killing performance and the number of wasps with killing performance of >40% decreased however to 20.7% ± 0.8SE and 10%, respectively (F7). The increased fly survival and the decrease in killing performance after the F4 generation suggests that fly host fitness increased over time. This hampers comparison of between generation performances of the wasps, as the trait value of killing performance is partly influenced by the fitness of the host.

The response to selection was therefore investigated by comparing the slope between fly survival and generation in the presence and absence of parasitoids (Figure 3a). GLMM analysis indicated that the temporal trend in fly survival was best predicted by fitting “generation” as quadratic variable (linear vs. quadratic: *χ* ^2^ = 5.00, *p* < 0.01). The estimates of the regression lines confirmed that parasitoids significantly reduced fly survival (*β* = −0.68, SE = 0.04, *p* < 0.01), and fly survival (as quadratic term) increased over generations independent of treatment (*β* = 0.01, SE = 0.002, *p* < 0.01). There was however also a significant interaction between treatment (wasp presence/absence) and generation (GLMM, change in slope in presence *versus* absence of wasps, *β* = −0.004, SE = 0.001, *χ* ^2^ (1) = 11.53, *p* < 0.01), which reflects that fly survival in absence of wasps increased relatively more than in the presence of selected wasps. In other words, the proportion of flies killed increased slightly despite the improved survival rate of the host, and thus confirms an effect of selection.

##### Selected versus unselected lines

**FIGURE 4:**
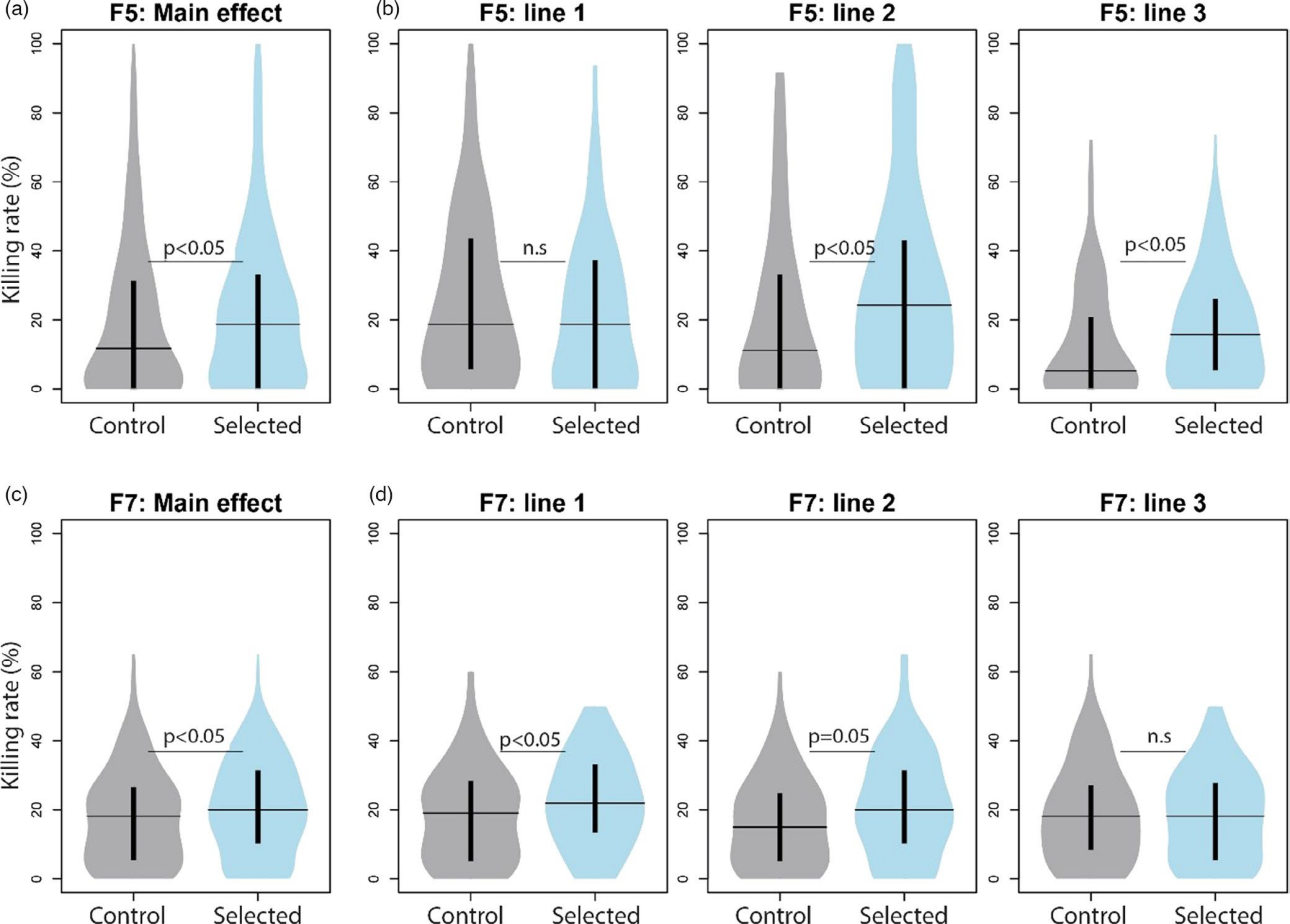
Killing rate of selected and unselected *Leptopilina* *heterotoma* wasps after five (a, b) and seven (c, d) generations of artificial selection, of the three replicate lines combined (*n* = 300) (a, c) and of each replicate line separately (*n* = 100) (b, d). Killing rate was calculated as the percentage of flies killed, adjusted for nonexposed fly mortality. Horizontal lines represent median killing rate and inner thick vertical line the interquartile range. Statistical differences by Tukey's post hoc test

After five generations of selection, wasps from the Selection lines killed significantly more flies compared to those from Control lines (Figure 4a) (GLMM *χ* ^2^ (1) = 4.08, *p* = 0.04). Analysis of each replicate line separately revealed that selected wasps of two replicate lines (S2 and S3) had a higher killing rate (GLMM line2: *χ* ^2^ (1) = 6.21, *p* = 0.01; line3: *χ* ^2^ (1) = 5.52, *p* = 0.02) and the other replicate (S1) performed similarly to its unselected control line (Figure 4b) (GLMM, *χ* ^2^ (1) = 1.29, *p* = 0.25). Similarly, after two more rounds of selection (F7), selected wasps killed significantly more flies compared to unselected wasps (Figure 4c) GLMM *χ* ^2^ (1) = 5.57, *p* = 0.02). Analysis of each replicate line separately demonstrated that selected wasps of two lines (S1, S2) (almost) significantly reduced *D. suzukii* survival relative to their unselected controls (GLMM line1: *χ* ^2^ (1) = 4.89, *p* = 0.03; line2: *χ* ^2^ (1) = 3.7, *p* = 0.054), but one line (S3) did not differ in killing performance (Figure 4d) (GLMM, *χ* ^2^ (1) = 0.004, *p* = 0.95). Yet, whereas selected and control wasps in the F5 generation had maximum trait values up to 100%, the highest killing rate in generation F7 was 63%, and the average killing rate had decreased with 2%. Taken together, selected wasps had increased killing rate compared to nonselected individuals, but the differences were relatively small. The decrease in host killing ability is in line with our observed increased host fitness, which reduced the response to selection. Unfortunately, due to logistic constraints, we were only able to measure and compare the unselected control wasp lines at generation 5 and 7.

##### Realized heritability

**FIGURE 5:**
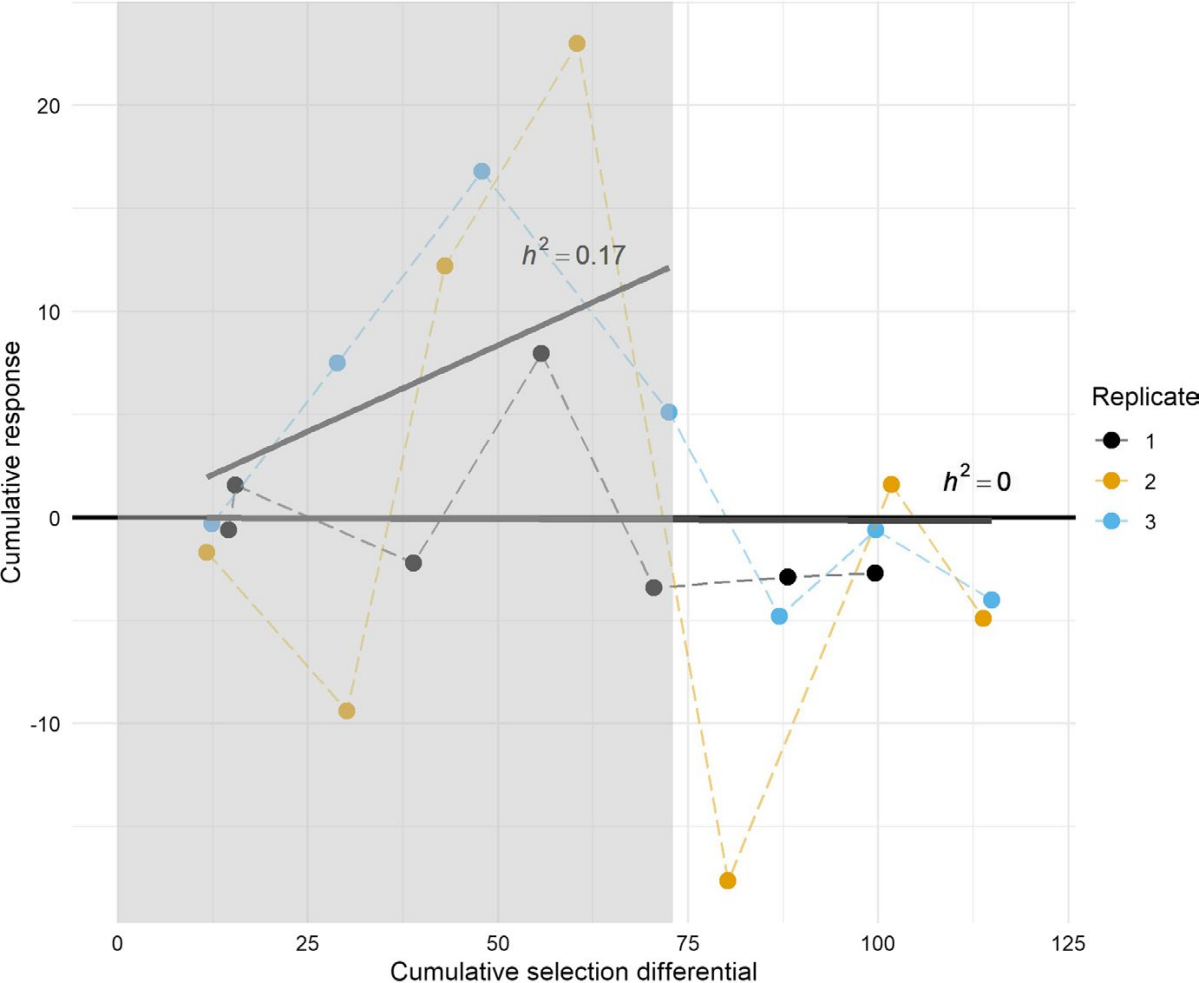
Realized heritability ($h^{2}$) of killing rate in artificial selection experiment of *Leptopilina* *heterotoma*. Cumulative response to selection (R) is plotted as function of the cumulative selection differential (S) for each replicate selected on increased killing rate for seven generations (dashed lines). The average realized heritability ($h^{2}$) was calculated as the slope of the linear regression between R and S until generation four and seven (straight lines). The gray area represents the cumulative response to selection until generation 4

Realized heritability ($h_{r}^{2}$) estimates (i.e., the response to selection as proportion of the amount of selection applied) fluctuated from zero to 0.310 between generations during the artificial selection experiment, but was not significantly different from zero after seven generations (*p* > 0.05) (Figure 5, Table 2). The average heritability was largest over the interval from P to F4, $h_{r}^{2}$ = 0.167, and differed significantly from zero (*p* = 0.01) (Figure 5, Table 2). Replicate lines also differed in response, the heritability estimate did significantly differ from zero for replicate line 3 after three generations of selection ($h_{r}^{2}$ = 0.31, *p* < 0.05), but not for the other replicates for any interval.

**TABLE 2:** Realized heritability estimates ($h_{r}^{2}$) for killing rate calculated as the slope of the linear regression between cumulative selection differential and response to selection over different generation intervals

	Realized heritability (std. error)	*F*‐statistic	Adj. *R* ^2^	*p*‐value
Average				
P‐F3	0.143 (0.081)	*F*_1,8_ = 3.21	0.1973	0.111
P‐F4	0.167 (0.053)*	*F*_1,11_ = 9.69	0.420	0.010
P‐F5	0.033 (0.052)	*F*_1,14_ = 0.40	−0.041	0.536
P‐F6	0.017 (0.036)	*F*_1,17_ = 0.45	−0.046	0.648
P‐F7	−0.001 (0.027)	*F*_1,20_ = 0.001	−0.050	0.961
Replicate 1				
P‐F3	−0.036 (0.036)	*F*_1,2_ = 0.96	−0.012	0.430
P‐F4	**0.074 (0.053)**	*F*_1,3_ = 1.89	0.182	0.263
P‐F5	0.013 (0.045)	*F*_1,4_ = 0.09	−0.223	0.782
P‐F6	−0.007 (0.032)	*F*_1,5_ = 0.05	−0.189	0.838
P‐F7	−0.014 (0.023)	*F*_1,6_ = 0.36	−0.101	0.572
Replicate 2				
P‐F3	0.077 (0.196)	*F*_1,2_ = 0.15	−0.393	0.734
P‐F4	**0.246 (0.137)**	*F*_1,3_ = 3.20	0.354	0.172
P‐F5	0.015 (0.144)	*F*_1,4_ = 0.01	−0.246	0.921
P‐F6	0.015 (0.096)	*F*_1,5_ = 0.02	−0.193	0.879
P‐F7	−0.005 (0.071)	*F*_1,6_ = 0.01	−0.165	0.941
Replicate 3				
P‐F3	0.310 (0.059)*	*F*_1,2_ = 27.30	0.897	0.035
P‐F4	0.162 (0.073)	*F*_1,3_ = 4.86	0.491	0.115
P‐F5	0.060 (0.071)	*F*_1,4_ = 0.71	−0.062	0.447
P‐F6	0.035 (0.052)	*F*_1,5_ = 0.45	−0.102	0.534
P‐F7	0.011 (0.041)	*F*_1,6_ = 0.08	−0.151	0.790

Note

Asterisk indicates whether the slope of the regression significantly differed from zero (*p* < 0.05), and bold indicates the highest heritability values of each replicate line.

#### 3.3.2 Correlated responses to selection

##### Attack rate

**FIGURE 6:**
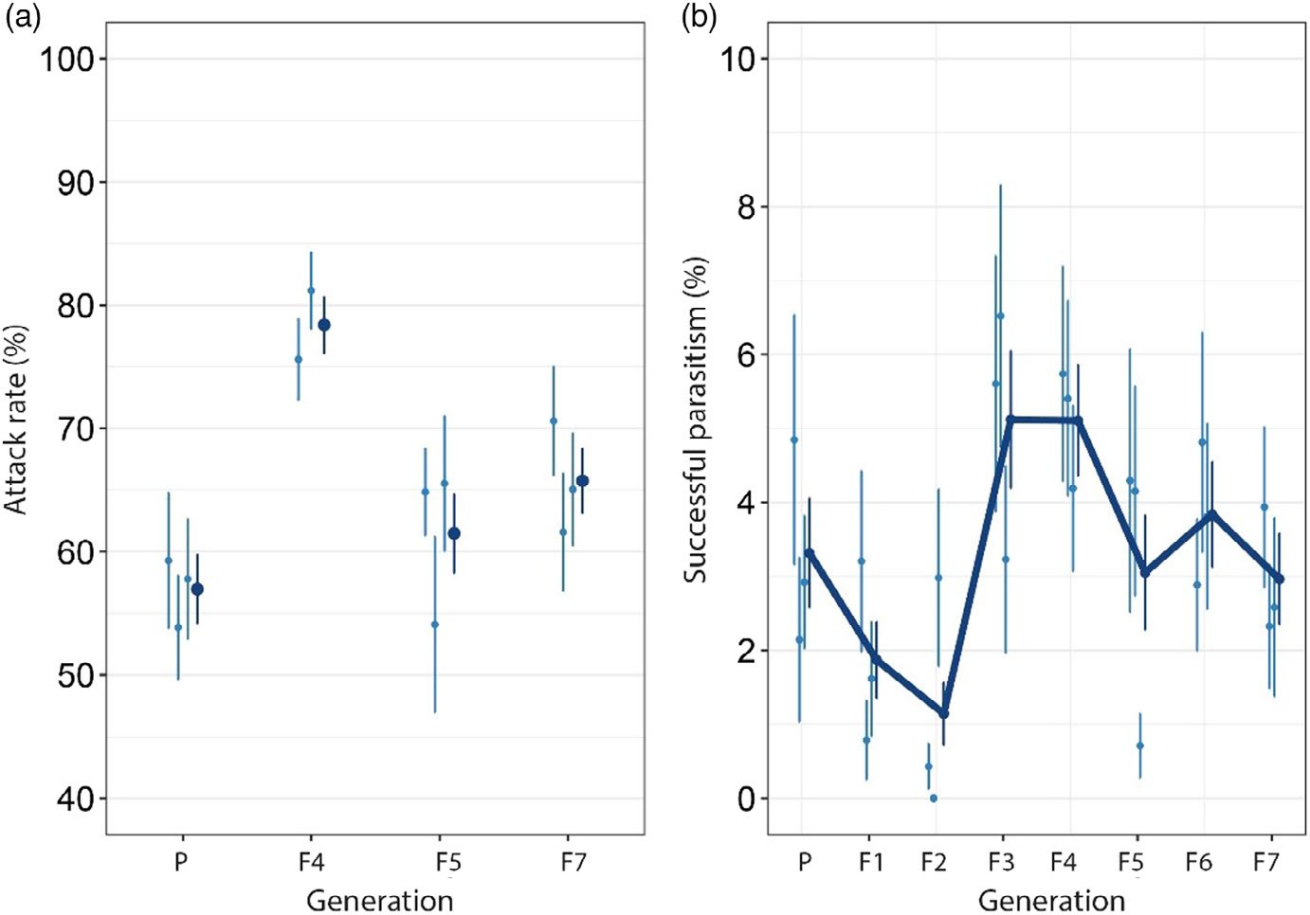
Correlated responses to selection for killing rate in *Leptopilina* *heterotoma*: (a) attack rate and (b) successful parasitism averaged over the three selection lines (dark blue), and of each selection separately (light blue dots). The average attack rate was calculated as the percentage of flies killed plus flies that survived but with encapsulated wasp egg. Successful parasitism was calculated as the percentage of killed flies that yielded offspring. Error bars represents standard errors of the mean. Note that sample size of the attack rate in the F4 was reduced (*n* = 60), as collected flies from one selection line were lost

Attack rate (i.e., the percentage of flies killed plus flies that survived but with encapsulated wasp egg) was calculated from a random sample (*n* = 30) of each replicate line at the start of selection (P) and in generations F4, F5, and F7. Wasps successfully attacked 64.5% ± 1.47 SE flies (all data combined), and 37.0% ± 1.50 SE of the attacked flies were killed (i.e., lethal attack rate). There was a near significant systematic increase in attack rate over time (Figure 6a) (GLMM, *β* = 0.06, SE = 0.035, *χ* ^2^ (1) = 3.68, *p* = 0.051) and generation had a significant effect when taken as categorical variable (GLMM, *χ* ^2^ (3) = 17.20, *p* < 0.001). The percentage of attacked flies was highest in the F4 generation compared to unselected wasps at the start (P) and the F5 and F7 generations (Tukey's test, *p* < 0.001). Generation did not have a significant effect on the proportion of attacked flies that were killed when taken as continuous explanatory variable (GLMM, *χ* ^2^ (1) = 2.94, *p* = 0.09) nor as categorical variable (GLMM, *χ* ^2^ (3) = 6.59, *p* = 0.09).

**FIGURE 7:**
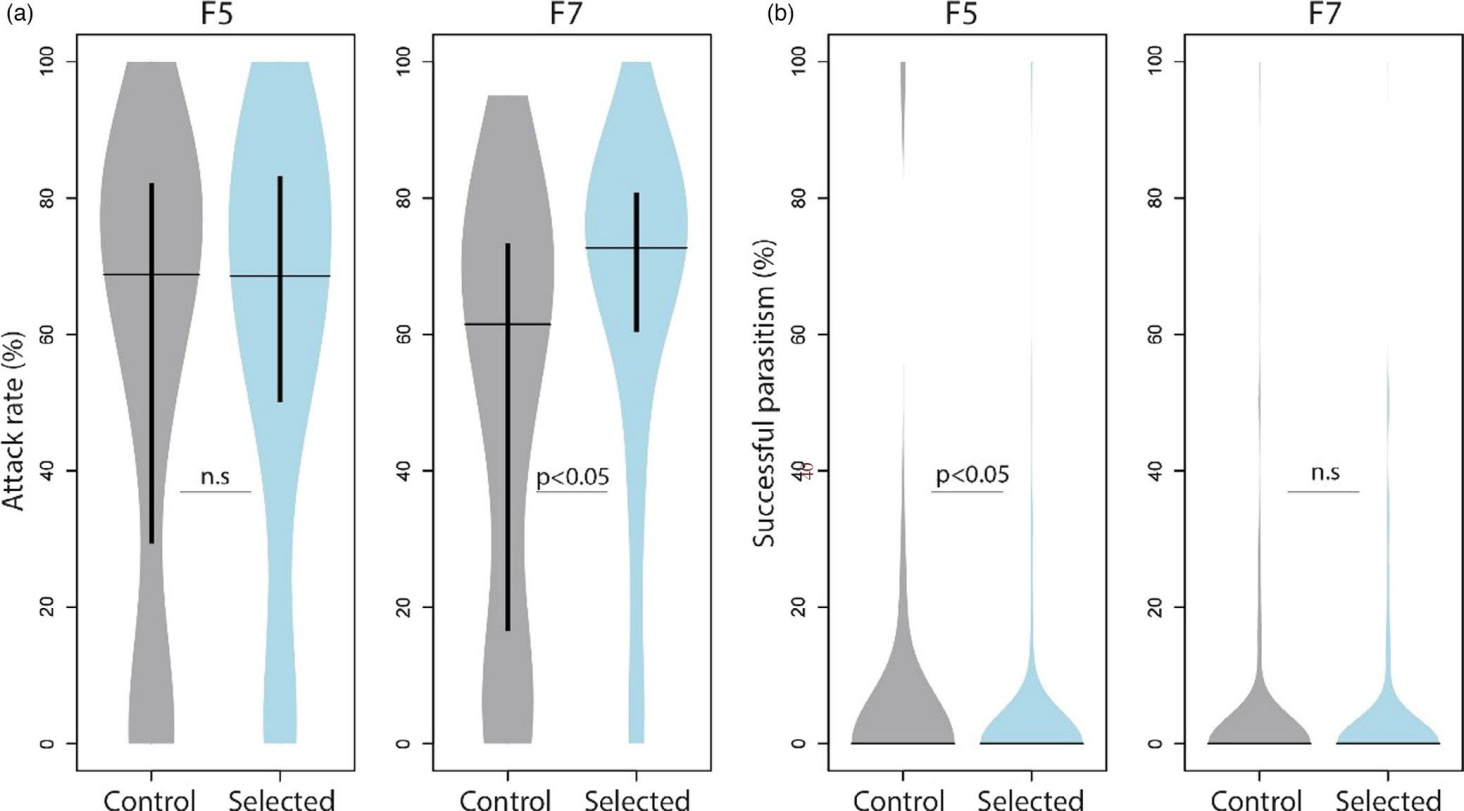
Correlated response to selection for killing rate in *Leptopilina* *heterotoma*. Attack rate (a) and Reproductive success (b) of selected and unselected wasps after five (F5) and seven (F7) generations of selection on killing performance. Attack rate was calculated as the percentage of flies killed, corrected for mortality in nonexposed flies, plus those that were parasitized but survived as measured from encapsulated parasitoid eggs (*n* = 90). Reproductive success was calculated as the percentage of killed flies that yielded offspring (*n* = 300). Horizontal lines represent median killing performance and inner thick vertical line the interquartile range

There was no difference in attack rate between selected and unselected wasps in the F5 generation (Figure 7) (GLMM, *χ* ^2^ (1) = 1.57, *p* = 0.21), neither in lethal attack rate (Control lines: 30.0% ± 30.2 SE, Selection lines: 30.6% ± 27.8, GLMM, *χ* ^2^ (1) = 1.07, *p* = 0.30). However, after seven generations of selection (F7), selected wasps had a significantly higher attack rate (GLMM, *χ* ^2^ (1) = 12.41, *p* < 0.001), though the proportion of killed flies was again similar (Control lines: 34.7% ± 29.5 SE, Selection lines: 29.6% ± 21.6 SE, GLMM, *χ* ^2^ (1) = 0.29, *p* = 0.59). Selection on killing performance thus seemed to have resulted in an increased attack rate, but wasps did not become more efficient in host killing as there was no clear effect on lethal attack rate. Note, however, that attack efficiency could also be confounded by the increased host fitness over the course of the experiment.

##### Reproductive success

Taking all generations into account, 13% (273/2381) of the wasps produced at least one offspring in *D. suzukii*. The majority, 82.8%, produced one offspring (*n* = 226) in a batch of 25 larvae, and only 12% produced two (*n* = 34), 4% three (*n* = 10), 0.7% four (*n* = 2), and one female produced five offspring. We tested whether killing rate was related to reproductive success, that is, whether those wasps able to reproduce had a higher killing activity compared to those that were not able to reproduce. Killing rate was compared between individuals that killed at least one host that resulted in one parasitoid offspring (*n* = 266), to those that also killed at least one host (killing rate > 0), but did not reproduce (*n* = 1652). This revealed no difference in killing performance between wasps that were able to reproduce and those that were not (32.8% ± 1.19 SE, 31.2% ± 0.48 SE, respectively, GLMM, *χ* ^2^ (1) = 0.583, *p* = 0.45).

Successful parasitism (i.e., the percentage of killed flies that yielded wasp offspring) differed between generations (Figure 6b) (GLMM, *χ* ^2^ (7) = 26.06, *p* < 0.001): it was highest in the F3 and F4 generations compared to first generations P‐F2 and last generations F5–F7. Reproductive success in the first two generations (P‐F2) and last two generations (F5–F7) was quite similar. This could correspond with the unfit host conditions during the first generations thereby decreasing their “quality” to support offspring survival, and the increased host fitness and thereby immune resistance in the latter generations. Comparison of selected and unselected wasps demonstrated that unselected wasps had a larger reproductive success in the F5 generation. There were slightly more individuals able to reproduce (Control lines: 13.1%, Selection lines: 8.3%, GLMM, *χ* ^2^ (1) = 3.64, *p* = 0.056), and unselected wasps had a significant higher successful parasitism rate (6.3%) compared to selected ones (3.1%) (Figure 7b) (GLMM, *χ* ^2^ (1) = 4.85, *p* = 0.03). After seven generations of selection, the number of wasps that successfully reproduced did not differ between selected (10.9%) and unselected wasps (9.1%) (GLMM, *χ* ^2^ (1) = 0.52, *p* = 0.47). Also, successful parasitism did not differ between selected and unselected wasp lines (C: 2.9%, S: 3.0%, GLMM, *χ* ^2^ (1) = 0.012, *p* = 0.91). Mechanisms of increased host killing ability thus do not seem to increase the probability of offspring survival. In addition, reproductive success seems largely determined by host fitness as indicated by significant differences between generations in relation to fly fitness.

### 3.4 Repeated selection on reproductive success

Over the course of the eight generations (P‐F7) of selective breeding for host‐killing, wasp offspring that emerged from *D. suzukii* were collected and used to set up a new line (Reproducers, or R1). In total, 102 females were collected that successfully emerged from *D. suzukii* during the course of the experiment. The performance tests of R1 females on *D. suzukii* resulted in 41 female offspring (R2). Selection of the second generation of successful reproducers resulted in 11 females that emerged from *D. suzukii* (R3). Parasitization performances of the third generation of reproducers (R3) were compared to the genetically diverse stock population (P), replicate 2 of the selection on killing (S2), as this line showed the largest and most consistent response to selection, and its control line (C2) (*n* = 25). Overall, parasitoids reduced fly survival (GLMM, *β* = −0.84, *χ* ^2^ (1) = 10.68, *p* < 0.001), but parasitoids with a history of developing on *D. suzukii* did not have higher killing rate (GLMM, *χ* ^2^ (3) = 6.65, *p* = 0.08). In fact, the R3 and the unselected wasp line (C2) killed slightly fewer flies compared to the wasp line selected for host killing (S2) and the diverse stock population (P) (R3: 16.7% ± 3.48 SE, C2: 15.4% ± 2.55 SE, S2: 22.9% ± 2.57 SE, P: 21.9% ± 2.53 SE). None of the wasps were able to reproduce on *D. suzukii*. Hence wasps selected on host killing and reproductive success did not differ in reproductive success on *D. suzukii*.

## 4 DISCUSSION

We investigated whether the invasive pest *D. suzukii* can become a novel host for the native parasitoid *L. heterotoma* by focusing on four traits reflecting sequential steps of the parasitization process: (1) attack rate, (2) killing rate, (3) lethal attack rate, and (4) successful offspring development (successful parasitism). Our study revealed that *L. heterotoma* exhibits significant heritable variation in attack rate ($h^{2}=0.44)$ and host killing ($h^{2}=0.28)$, lethal attack rate ($h^{2}=0.61$) but not in offspring survival ($h^{2}=0.0)$. Contrary to what we expected, the response to selection after seven generations of directional selection on increased killing rate relative to the amount of selection applied, realized heritability ($h_{r}^{2}$), was zero. Moreover, selection yielded increased killing rate in selected wasps compared to nonselected individuals at generation five and seven, but the differences were relatively small (2%–8%) and were inconsistent among replicates, indicating that these differences could be due to either selection or drift. Yet, realized heritability after the first four generations was significant, $h_{r}^{2}$ = 0.17, and similar to our heritability estimate of the half‐sib analysis, we did find a consistent and strong correlative response in the attack rate (15% improvement relative to control lines). Hence, selection resulted in an increased attack rate rather than nonreproductive host killing after 7 generations of selection. The decline in response to selection is in line with our observed increased *D. suzukii* fitness over the course of the tested generations, which might have reduced the response to selection. Selection did not improve successful parasitism, and parasitoid offspring that emerged from *D. suzukii* did not exhibit enhanced killing and reproduction, suggesting that the ability to overcome host defenses did not directly affect the success rate at the intermediate steps in the parasitization process.

### 4.1 Factors that reduce response to selection

The increase in host‐killing rate in response to artificial selection was minor. Several factors could have reduced the magnitude of response to selection. An important factor is variation in phenotypic traits of the host: the likelihood of being found and surviving parasitoid attack (Kruitwagen et al., 2018). Given that over the course of the selection experiment (1 year) *D. suzukii* survival increased by 30% and the stock population had only been established several months before initiation of the experiment, laboratory adaptation in the host strain seems to have occurred. Following the resource allocation theory, resources invested in life history traits such as growth and reproduction can come at the expense of other energetic costly traits, like the maintenance and deployment of immune defenses (Rauw, 2012; Schwenke et al., 2016). These life‐history trade‐offs can become more pronounced under environmental stress, such as food limitation and desiccation (Hoang, 2001; Moret & Schmid‐Hempel, 2000). Hence, when the *D. suzukii* stock population became more adapted to the laboratory, more resources might have become available to allocate to immunity, making the flies more resistant to parasitoid attack. The low improvement in killing performance after seven generations of selection could then be explained by the relative faster evolution of the hosts compared to the parasitoids' killing rate. This slowed down the response to selection, albeit it was still detectable, and even decreased the wasps' ability to kill the flies despite evolution of killing rate. Alternatively, *D. suzukii* might have evolved generic counter defenses to parasitoids (e.g., increased hemocyte quantity or quality) (Gerritsma et al., 2013; Kacsoh & Schlenke, 2012; Wertheim et al., 2011), or specific defenses (e.g., against venom components of *L. heterotoma*) (Colinet et al., 2013; Fellowes et al., 1999) over the course of the experiment. However, flies that survived parasitoid attack were never placed back in the stock population, making evolution of resistance to parasitoid attack unlikely.

Besides the increased *D. suzukii* host fitness, several other genetic and environmental factors could have contributed to the low response to selection. Firstly, being a complex behavioral trait, killing performance likely depends on many genes. For example, virus like particles (Cavigliasso et al., 2019; Mathe‐Hubert et al., 2019; Poirié et al., 2009), ovipositor morphology (van Lenteren et al., 1998) and neuro‐sensory characteristics (Ruschioni et al., 2015; van Lenteren et al., 2007) have been shown to be important for parasitization in *L. heterotoma* and might be subject to evolutionary change. Therefore, it would take multiple generations to establish a shift in allele frequencies (Falconer & Mackay, 1996). Secondly, evolutionary change might have been limited due to a decrease in additive genetic variance and/or loss of alleles due to genetic drift over successive generations of selection. However, this is assumed to become more pronounced in long‐term selection experiments and under strong selection or bottlenecks (Barton & Partridge, 2000; Careau et al., 2013; Falconer & Mackay, 1996). Thirdly, our estimate of heritability of killing rate in the base population was $h^{2}=0.28$, meaning that phenotypic variance is also influenced for a substantial part by nonadditive genetic variation, reducing the potential for selection. Similarly, Henter (1995), estimated that variation in successful parasitism rate was attributed for 57% to environmental effects in the aphid parasitoid *Aphidius ervi*. Natal host quality (*D. melanogaster*) (A. Kruitwagen et al., unpublished results; Harvey, 2000; Ris et al., 2004; Rosenheim & Rosen, 1992), and the wasps' nutritional status (Ellers et al., 1998; Jervis et al., 2008) could also have influenced the wasp's ability and willingness to parasitize. Although every attempt was made to maintain constant conditions, we cannot exclude any unintentional environmental variation. This underlines the importance of understanding the sources of variation to increase the response to selection when attempting to genetically improve natural enemies for biocontrol (Kruitwagen et al., 2018).

### 4.2 Mechanisms underlying the evolution of nonreproductive host mortality

Our findings are in line with previous reports on the widespread nature of nonreproductive host mortality in host–parasitoid systems (Abram et al., 2016, 2019; Heimpel et al., 2003), including the *L. heterotoma*–*D. suzukii* system (Chabert et al., 2012; Iacovone et al., 2018; Kacsoh & Schlenke, 2012; Mazzetto et al., 2016). The larvae of koinobiont parasitoids, like *L. heterotoma*, feed from the developing host larvae until the parasitoid reaches the pupal stage, after which the host dies (Fleury et al., 2009). However, when the host dies too soon the parasitoid is unable to develop and survive (Rizki & Rizki, 1990). This raises the question which mechanism(s) underlie the induction of nonreproductive host mortality. Generalist species like *L. heterotoma* might have an “opportunistic” strategy reflected in their low threshold for host species acceptance, readily parasitizing novel hosts like *D. suzukii*, combined with a high egg load (i.e., eggs are less “costly”). Alternatively, they might be unable to distinguish between host (habitat) cues of suitable hosts and *D. suzukii* that predict offspring survival. Consequently, nonreproductive host mortality might be a “by‐product” of selection for parasitizing on other hosts. Evolution of nonreproductive host‐killing and the evolutionary consequences of these maladaptive host choices have not been studied extensively. Our findings provide new avenues for such efforts to understand and predict the evolution of this trait and how this influences host range evolution.

Interestingly, selection on host killing resulted in a correlated response in attack rate, but wasps did not become more efficient in host killing. Perhaps wasps increased their search time, acceptance rate and/or ability to recognize hosts' species presence as a result of selection. An increase in lethal attack rate could then have come about by not only attacking more hosts, but also by attacking the same host multiple times and thus further enhancing their damage. Activity level in *L. heterotoma* (Fleury et al., 1995) and host selection in the *Asobara* *tabida*–*Drosophila* system (Mollema, 1990; Rolff & Kraaijeveld, 2001) were also found to be partly determined by genetic effects, suggesting that these first steps of parasitization process (host finding–acceptance) can evolve relative quickly in response to selection. The lack of response in lethal attack rate in our experiment was surprising as European populations expressed major differences (Figure 1c). Moreover, lethal attack rate had a relatively high heritability $h^{2}=0.61$. Considering that the lethal attack rate was slightly higher in the F4 compared to the start of the experiment, but then decreased in the F5 and F7, this trait is likely more sensitive to the rise in fitness of *D. suzukii* than the attack rate. As a consequence, such a difference in environmental sensitivity of different parasitization parameters can facilitate maintenance of variation for the outcome of host‐parasite interactions (Duneau et al., 2011; Ebert et al., 2016; Hall et al., 2017). More research is needed to identify specific “traits” underlying host killing and their sources of variation to understand what could constrain or favor formation of novel host‐parasitoid associations.

### 4.3 Host killing as intermediate step to a complete host shift/expansion

The relationship between host choice and offspring survival is fundamental for parasitoids' fitness, host‐range, and population persistence (Henry et al., 2005; Jaenike, 1978; Thompson, 1988). Maladaptive host choices, that is attacking hosts that do not support offspring development, can impose costs (e.g., time and resources) on the individual and lead to an evolutionary trap when females' are not able to discriminate between suitable and unsuitable host species (Abram et al., 2014, 2019; Schlaepfer et al., 2005). We therefore investigated the severity of the *D. suzukii* “trap” for *L. heterotoma* to see whether females are able to overcome the host defense barrier. We found however no positive relationship between host‐killing and reproductive success and no indications of genetic variation in reproductive success. This suggest that host‐killing is not an effective step to a complete host‐shift/expansion in the short term in this experimental system. Although we did not find indications of host‐shift/host‐range expansion evolution, this might occur when a genetic variant appears that is able to evade host‐immunity and survive inside the host. Future sampling and testing of populations in space and time might reveal how natural enemies evolve to the novel host, i.e., whether a novel biotic interaction arises, or alternatively, whether they evolve to avoid this host.

The evolution of a generalist strategy can be restricted by trade‐offs: adaptation to one resource can decrease fitness in another (Agrawal, 2000; Elena & Lenski, 2003; Fry, 1990; Henry et al., 2008; Jaenike, 1990; Via & Hawthorne, 2002). In order to reproduce on *D. suzukii*, parasitoids might need another parasitization strategy then on their native hosts like *D. melanogaster*. As such, a complete host shift might have been hampered in our selection experiment as parasitoids were cultured on *D. melanogaster*. Interestingly, unselected wasps showed a slightly larger reproductive success in the F5 compared to selected ones and selection on reproductive success did not enhance either killing or reproductive success rates. So, evolution of host‐range might also have been constrained by functional correlations between traits within the same host: The parasitoids' host‐killing mechanism might not always promote offspring survival. For example, superparasitism (i.e., laying multiple eggs in the same host) or high venom quantity might kill the host before full development of the parasitoids. Selection however did not seem to affect exploitation success when the wasps were provided with *D. melanogaster* as there was no large change observed in reproductive success during and after selective breeding (>70% successful parasitism) and comparison of selected and control lines in the F7 (*n* = 65) yielded no significant differences in killing rate of *D. melanogaster* (GLMM, *χ* ^2^ (1) = 0.807, *p* = 0.37) and successful parasitism (GLMM, *χ* ^2^ (1) = 0.807, *p* = 0.1).

### 4.4 Implications of nonreproductive host‐killing for biocontrol

Despite the widespread nature of nonreproductive effects in host‐parasitoid systems, they are underappreciated for biocontrol, as the main focus is often on reproductive success (Abram et al., 2016, 2019). Recent findings, however, indicate that these effects can be an important mechanism that augment the biocontrol performance of parasitoids (Huang et al., 2017; Kaser et al., 2018; Münster‐Swendsen, 2002). Our study adds that nonreproductive host killing has a genetic component, but is also sensitive to environmental conditions resulting in fluctuating heritability. The presence of significant heritable variation thus does not guarantee improvement by artificial selection, this can only occur when stable environmental conditions (e.g., host fitness) over the course of selection and high accuracy of selection can be achieved. For example, if environmental conditions, intensity of selection and heritability would have remained constant over generations in our experiment, following the breeders' equation ($R=i\sigma_{a}\surd h^{2}$) (Falconer & Mackay, 1996), it would have taken about seven generations to increase killing rate to 97.25% (assuming selection intensity(i) of 0.79 (Wricke & Weber, 1986), additive genetic variance of $\sigma_{a}^{2}=\surd 1.08$ and $h^{2}=0.28$).

To understand and predict the ability of *L. heterotoma* to regulate *D. suzukii* population growth under field conditions and agricultural settings (e.g., greenhouses or orchards), more research is needed in parasitoids' ability to find hosts, their attack rate in fresh fruits, and its host species preference in the field. In addition, following our observation that *D. suzukii* resistance to parasitoid attack can vary, biological control would benefit from investigating the immune resistance under different field conditions. Ultimately, for immediate within‐generation control, parasitoids displaying nonreproductive host killing could be used in pest management by inundative application (i.e., the release of large numbers of natural enemies for achieving a rapid effect), as they can cause substantial mortality in host larvae. For multi‐generational effects, they can in theory be supplied by using a banker system, providing them with alternative (nonharmful) hosts to support the population growth of the biocontrol agent (e.g., providing a susceptible host like *D. melanogaster* as a reservoir in the case of *L. heterotoma*). However, such banker system or continued inundative releases might not be economically feasible. As such, unless the attack and host‐killing ability of *L. heterotoma* in the field are sufficient to suppress *D. suzukii* population levels under the economic threshold level, this study demonstrates that genetic improvement of *L. heterotoma* does not have enough promise for effective biocontrol of *D. suzukii*.

Interestingly, exotic natural enemies of the pest are sometimes accidently introduced in the pests' invaded range (e.g. Frewin et al., 2010; Stahl et al., 2019), including enemies of *D. suzukii* (Abram et al., 2020): two larval parasitoids specialized on *D. suzukii* have been detected in Canada (*Leptopilina japonica*, and *Ganaspis brasiliensis*). Hence, such accidental introductions can thus create new opportunities for biological control, circumventing federal regulations for planned importation and release of exotic species. Yet, their quality and potential biodiversity risks should be evaluated before inundative/inoculative release.

## 5 CONCLUSION

Our results provide an example of how evolutionary principles can be applied to optimize performance of native species for biological control. We consider the evolution of host‐range as a stepwise process and assessed whether intermediate steps can be selected for. This adds to the growing body of evidence that natural enemies can evolve to overcome host defenses and be optimized for biocontrol (e.g. Dennis et al., 2017; Henry et al., 2008; Kraaijeveld et al., 2001; Lirakis & Magalhães, 2019; Rossbacher & Vorburger, 2020). Even though we did not observe a major effect of artificial selection in nonreproductive host killing, we showed that wasps exhibited genetic variation in nonreproductive host killing, which can influence host population dynamics (Kaser et al., 2018) and thus be an asset for biological control. Host specific factors, such as resistance and condition, will be crucial for the likelihood of adaptation in natural populations of natural enemies by affecting the reproductive success and thus parasitoid fitness. Empirical and theoretical studies are required linking traits underlying multihost parasitization and direction of selection pressures in nature to understand and predict the response of native natural enemies to novel invasive species and their eco‐evolutionary consequences.

## CONFLICTS OF INTEREST

The authors have no conflicts of interest to disclose.

## Data Availability

The data can be accessed at the Dryad Digital Repository using the following link: doi:10.5061/dryad.dr7sqv9xx [Correction added on 10 June 2021, after first online publication: Data Availability Statement has been updated in this version]

## References

[eva13252-bib-0001] Abram, P., Brodeur, J., Burte, V., & Boivin, G. (2016). Parasitoid‐induced host egg abortion: An underappreciated component of biological control services provided by egg parasitoids. Biological Control, 98, 52–60.

[eva13252-bib-0002] Abram, P., Brodeur, J., Urbaneja, A., & Tena, A. (2019). Nonreproductive effects of insect parasitoids on their hosts. Annual Review of Entomology, 64, 259–276. 10.1146/annurev-ento-011118-111753 30312554

[eva13252-bib-0003] Abram, P., Gariepy, T., Boivin, G., & Brodeur, J. (2014). An invasive stink bug as an evolutionary trap for an indigenous egg parasitoid. Biological Invasions, 16(7), 1387–1395.

[eva13252-bib-0004] Abram, P. K., McPherson, A. E., Kula, R., Hueppelsheuser, T., Thiessen, J., Perlman, S. J., Curtis, C. I., Fraser, J. L., Tam, J., Carrillo, J., Gates, M., Scheffer, S., Lewis, M., & Buffington, M. (2020). New records of Leptopilina, Ganaspis, and Asobara species associated with *Drosophila suzukii* in North America, including detections of *L. japonica* and *G. brasiliensis* . Journal of Hymenoptera Research, 78, 1–17.

[eva13252-bib-0005] Agrawal, A. F. (2000). Host‐range evolution: Adaptation and trade‐offs in fitness of mites on alternative hosts. Ecology, 81(2), 500–508.

[eva13252-bib-0006] Agrawal, A. F., & Lively, C. M. (2003). Modelling infection as a two‐step process combining gene‐for‐gene and matching‐allele genetics. Proceedings of the Royal Society of London. Series B: Biological Sciences, 270(1512), 323–334. 12614583 10.1098/rspb.2002.2193 PMC1691240

[eva13252-bib-0007] Asgari, S., & Rivers, D. B. (2011). Venom proteins from endoparasitoid wasps and their role in host‐parasite interactions. Annual Review of Entomology, 56, 313–335. 10.1146/annurev-ento-120709-144849 20822448

[eva13252-bib-0008] Ashley, M. V., Willson, M. F., Pergams, O. R., O'Dowd, D. J., Gende, S. M., & Brown, J. S. (2003). Evolutionarily enlightened management. Biological Conservation, 111(2), 115–123.

[eva13252-bib-0009] Atallah, J., Teixeira, L., Salazar, R., Zaragoza, G., & Kopp, A. (2014). The making of a pest: The evolution of a fruit‐penetrating ovipositor in *Drosophila suzukii* and related species. Proceedings of the Royal Society B: Biological Sciences, 281(1781), 20132840. 10.1098/rspb.2013.2840 PMC3953835 24573846

[eva13252-bib-0010] Barton, N., & Partridge, L. (2000). Limits to natural selection. BioEssays, 22(12), 1075–1084. 11084623 10.1002/1521-1878(200012)22:12<1075::AID-BIES5>3.0.CO;2-M

[eva13252-bib-0011] Bates, D., Mächler, M., Bolker, B., & Walker, S. (2014). Fitting linear mixed‐effects models using lme4. arXiv preprint arXiv:1406.5823.

[eva13252-bib-0012] Calabria, G., Máca, J., Bächli, G., Serra, L., & Pascual, M. (2012). First records of the potential pest species *Drosophila suzukii* (Diptera: Drosophilidae) in Europe. Journal of Applied Entomology, 136(1–2), 139–147.

[eva13252-bib-0013] Careau, V., Wolak, M. E., Carter, P. A., & Garland, T. Jr (2013). Limits to behavioral evolution: The quantitative genetics of a complex trait under directional selection. Evolution, 67(11), 3102–3119. 24151996 10.1111/evo.12200

[eva13252-bib-0014] Carlsson, N. O., Sarnelle, O., & Strayer, D. L. (2009). Native predators and exotic prey–An acquired taste? Frontiers in Ecology and the Environment, 7(10), 525–532.

[eva13252-bib-0015] Carroll, S. P. (2011). Conciliation biology: The eco‐evolutionary management of permanently invaded biotic systems. Evolutionary Applications, 4(2), 184–199. 25567967 10.1111/j.1752-4571.2010.00180.x PMC3352563

[eva13252-bib-0016] Carroll, S. P., Loye, J. E., Dingle, H., Mathieson, M., Famula, T. R., & Zalucki, M. P. (2005). And the beak shall inherit–evolution in response to invasion. Ecology Letters, 8(9), 944–951. 10.1111/j.1461-0248.2005.00800.x 34517679

[eva13252-bib-0017] Cavigliasso, F., Mathé‐Hubert, H., Kremmer, L., Rebuf, C., Gatti, J.‐L., Malausa, T., Colinet, D., & Poirié, M. (2019). Rapid and differential evolution of the venom composition of a parasitoid wasp depending on the host strain. Toxins, 11(11), 629. 10.3390/toxins11110629 PMC6891688 31671900

[eva13252-bib-0018] Chabert, S., Allemand, R., Poyet, M., Eslin, P., & Gibert, P. (2012). Ability of European parasitoids (Hymenoptera) to control a new invasive Asiatic pest, *Drosophila suzukii* . Biological Control, 63(1), 40–47.

[eva13252-bib-0019] Cock, M. J. W., van Lenteren, J. C., Brodeur, J., Barratt, B. I. P., Bigler, F., Bolckmans, K., Cônsoli, F. L., Haas, F., Mason, P. G., & Parra, J. R. P. (2010). Do new access and benefit sharing procedures under the convention on biological diversity threaten the future of biological control? BioControl, 55(2), 199–218.

[eva13252-bib-0020] Colautti, R. I., Ricciardi, A., Grigorovich, I. A., & MacIsaac, H. J. (2004). Is invasion success explained by the enemy release hypothesis? Ecology Letters, 7(8), 721–733.

[eva13252-bib-0021] Colinet, D., Deleury, E., Anselme, C., Cazes, D., Poulain, J., Azema‐Dossat, C., Belghazi, M., Gatti, J.‐L., & Poirié, M. (2013). Extensive inter‐and intraspecific venom variation in closely related parasites targeting the same host: The case of Leptopilina parasitoids of Drosophila. Insect Biochemistry and Molecular Biology, 43(7), 601–611. 23557852 10.1016/j.ibmb.2013.03.010

[eva13252-bib-0022] De Clercq, P., Mason, P. G., & Babendreier, D. (2011). Benefits and risks of exotic biological control agents. BioControl, 56(4), 681–698.

[eva13252-bib-0023] De Ros, G., Conci, S., Pantezzi, T., & Savini, G. (2015). The economic impact of invasive pest *Drosophila suzukii* on berry production in the Province of Trento. Italy. Journal of Berry Research, 5(2), 89–96.

[eva13252-bib-0024] de Villemereuil, P. (2012). Estimation of a biological trait heritability using the animal model: How to use the MCMCglmm R package. Tutorial published online at: http://devillemereuil.legtux.org/wp‐content/uploads/2012/12/tuto_en.pdf

[eva13252-bib-0025] de Villemereuil, P., Gimenez, O., & Doligez, B. (2013). Comparing parent–offspring regression with frequentist and Bayesian animal models to estimate heritability in wild populations: A simulation study for Gaussian and binary traits. Methods in Ecology and Evolution, 4(3), 260–275.

[eva13252-bib-0026] Dennis, A. B., Patel, V., Oliver, K. M., & Vorburger, C. (2017). Parasitoid gene expression changes after adaptation to symbiont‐protected hosts. Evolution, 71(11), 2599–2617. 28841224 10.1111/evo.13333

[eva13252-bib-0027] Desurmont, G. A., Donoghue, M. J., Clement, W. L., & Agrawal, A. F. (2011). Evolutionary history predicts plant defense against an invasive pest. Proceedings of the National Academy of Sciences of the United States of America, 108(17), 7070–7074. 21482779 10.1073/pnas.1102891108 PMC3084082

[eva13252-bib-0028] DiTommaso, A., & Losey, J. E. (2003). Oviposition preference and larval performance of monarch butterflies (*Danaus plexippus*) on two invasive swallow‐wort species. Entomologia Experimentalis Et Applicata, 108(3), 205–209.

[eva13252-bib-0029] Duneau, D., Luijckx, P., Ben‐Ami, F., Laforsch, C., & Ebert, D. (2011). Resolving the infection process reveals striking differences in the contribution of environment, genetics and phylogeny to host‐parasite interactions. BMC Biology, 9(1), 11. 21342515 10.1186/1741-7007-9-11 PMC3052238

[eva13252-bib-0030] Ebert, D., Duneau, D., Hall, M. D., Luijckx, P., Andras, J. P., Du Pasquier, L., & Ben‐Ami, F. (2016). A population biology perspective on the stepwise infection process of the bacterial pathogen *Pasteuria ramosa* in Daphnia. In D. Rollinson & J. R. Stothard (Eds.), Advances in parasitology (Vol. 91, pp. 265–310). Elsevier. 10.1016/bs.apar.2015.10.001 27015951

[eva13252-bib-0031] Elena, S. F., & Lenski, R. E. (2003). Evolution experiments with microorganisms: The dynamics and genetic bases of adaptation. Nature Reviews Genetics, 4(6), 457–469. 10.1038/nrg1088 12776215

[eva13252-bib-0032] Ellers, J., van Alphen, J. J., & Sevenster, J. G. (1998). A field study of size–fitness relationships in the parasitoid *Asobara tabida* . Journal of Animal Ecology, 67(2), 318–324.

[eva13252-bib-0033] Falconer, D. S., & Mackay, T. F. C. (1996). Introduction to quantitative genetics (4th ed.). Longman.

[eva13252-bib-0034] Farnsworth, D., Hamby, K. A., Bolda, M., Goodhue, R. E., Williams, J. C., & Zalom, F. G. (2017). Economic analysis of revenue losses and control costs associated with the spotted wing drosophila, *Drosophila suzukii* (Matsumura), in the California raspberry industry. Pest Management Science, 73(6), 1083–1090. 27943618 10.1002/ps.4497

[eva13252-bib-0035] Fellowes, M., Kraaijeveld, A., & Godfray, H. (1999). Cross‐resistance following artificial selection for increased defense against parasitoids in *Drosophila melanogaster* . Evolution, 53(3), 966–972. 28565619 10.1111/j.1558-5646.1999.tb05391.x

[eva13252-bib-0036] Fleury, F., Allemand, R., Fouillet, P., & Boulétreau, M. (1995). Genetic variation in locomotor activity rhythm among populations of *Leptopilina heterotoma* (Hymenoptera: Eucoilidae), a larval parasitoid of Drosophila species. Behavior Genetics, 25(1), 81–89. 7755522 10.1007/BF02197245

[eva13252-bib-0037] Fleury, F., Gibert, P., Ris, N., & Allemand, R. (2009). Ecology and life history evolution of frugivorous Drosophila parasitoids. Advances in Parasitology, 70, 3–44. 19773065 10.1016/S0065-308X(09)70001-6

[eva13252-bib-0038] Fraimout, A., Debat, V., Fellous, S., Hufbauer, R. A., Foucaud, J., Pudlo, P., Marin, J.‐M., Price, D. K., Cattel, J., Chen, X., Deprá, M., Duyck, P. F., Guedot, C., Kenis, M., Kimura, M. T., Loeb, G., Loiseau, A., Martinez‐Sañudo, I., Pascual, M., … Estoup, A. (2017). Deciphering the routes of invasion of *Drosophila suzukii* by means of ABC random forest. Molecular Biology and Evolution, 34(4), 980–996. 28122970 10.1093/molbev/msx050 PMC5400373

[eva13252-bib-0039] Frewin, A. J., Xue, Y., Welsman, J. A., Broadbent, B. A., Schaafsma, A. W., & Hallett, R. H. (2010). Development and parasitism by *Aphelinus certus* (Hymenoptera: Aphelinidae), a parasitoid of *Aphis glycines* (Hemiptera: Aphididae). Environmental Entomology, 39(5), 1570–1578. 22546454 10.1603/EN09312

[eva13252-bib-0040] Fry, J. D. (1990). Trade‐offs in fitness on different hosts: Evidence from a selection experiment with a phytophagous mite. The American Naturalist, 136(5), 569–580.

[eva13252-bib-0041] Fry, J. D. (2003). Detecting ecological trade‐offs using selection experiments. Ecology, 84(7), 1672–1678.

[eva13252-bib-0042] Gandhi, K. J., & Herms, D. A. (2010). Direct and indirect effects of alien insect herbivores on ecological processes and interactions in forests of eastern North America. Biological Invasions, 12(2), 389–405.

[eva13252-bib-0043] Gerritsma, S., de Haan, A., van de Zande, L., & Wertheim, B. (2013). Natural variation in differentiated hemocytes is related to parasitoid resistance in *Drosophila melanogaster* . Journal of Insect Physiology, 59(2), 148–158. 23123513 10.1016/j.jinsphys.2012.09.017

[eva13252-bib-0044] Gross, P. (1993). Insect behavioral and morphological defenses against parasitoids. Annual Review of Entomology, 38(1), 251–273.

[eva13252-bib-0045] Hadfield, J. D. (2010). MCMC methods for multi‐response generalized linear mixed models: The MCMCglmm R package. Journal of Statistical Software, 33(2), 1–22.20808728

[eva13252-bib-0046] Hajek, A. E., Hurley, B. P., Kenis, M., Garnas, J. R., Bush, S. J., Wingfield, M. J., van Lenteren, J. C., & Cock, M. J. W. (2016). Exotic biological control agents: A solution or contribution to arthropod invasions? Biological Invasions, 18(4), 953–969.

[eva13252-bib-0047] Hall, M. D., Bento, G., & Ebert, D. (2017). The evolutionary consequences of stepwise infection processes. Trends in Ecology & Evolution, 32(8), 612–623. 28648806 10.1016/j.tree.2017.05.009

[eva13252-bib-0048] Harrison, X. A. (2015). A comparison of observation‐level random effect and Beta‐Binomial models for modelling overdispersion in Binomial data in ecology & evolution. PeerJ, 3, e1114. 26244118 10.7717/peerj.1114 PMC4517959

[eva13252-bib-0049] Harvey, J. A. (2000). Dynamic effects of parasitism by an endoparasitoid wasp on the development of two host species: Implications for host quality and parasitoid fitness. Ecological Entomology, 25(3), 267–278.

[eva13252-bib-0050] Hauser, M. (2011). A historic account of the invasion of *Drosophila suzukii* (Matsumura) (Diptera: Drosophilidae) in the continental United States, with remarks on their identification. Pest Management Science, 67(11), 1352–1357. 21898759 10.1002/ps.2265

[eva13252-bib-0051] Heimpel, G. E., Neuhauser, C., & Hoogendoorn, M. (2003). Effects of parasitoid fecundity and host resistance on indirect interactions among hosts sharing a parasitoid. Ecology Letters, 6(6), 556–566.

[eva13252-bib-0052] Henry, L. M., Gillespie, D. R., & Roitberg, B. D. (2005). Does mother really know best? Oviposition preference reduces reproductive performance in the generalist parasitoid *Aphidius ervi* . Entomologia Experimentalis Et Applicata, 116(3), 167–174.

[eva13252-bib-0053] Henry, L. M., May, N., Acheampong, S., Gillespie, D. R., & Roitberg, B. D. (2010). Host‐adapted parasitoids in biological control: Does source matter? Ecological Applications, 20(1), 242–250. 20349844 10.1890/08-1869.1

[eva13252-bib-0054] Henry, L. M., Roitberg, B. D., & Gillespie, D. R. (2008). Host‐range evolution in Aphidius parasitoids: Fidelity, virulence and fitness trade‐offs on an ancestral host. Evolution, 62(3), 689–699. 18182071 10.1111/j.1558-5646.2007.00316.x

[eva13252-bib-0055] Henter, H. J. (1995). The potential for coevolution in a host‐parasitoid system. II. Genetic variation within a population of wasps in the ability to parasitize an aphid host. Evolution, 49(3), 439–445. 28565084 10.1111/j.1558-5646.1995.tb02276.x

[eva13252-bib-0056] Hoang, A. (2001). Immune response to parasitism reduces resistance of *Drosophila melanogaster* to desiccation and starvation. Evolution, 55(11), 2353–2358. 11794793 10.1111/j.0014-3820.2001.tb00748.x

[eva13252-bib-0057] Hoy, M. A. (1986). Use of genetic improvement in biological control. Agriculture, Ecosystems & Environment, 15(2–3), 109–119.

[eva13252-bib-0058] Huang, J., Hua, H.‐Q., Wang, L.‐Y., Zhang, F., & Li, Y.‐X. (2017). Number of attacks by *Trichogramma dendrolimi* (Hymenoptera: Trichogrammatidae) affects the successful parasitism of *Ostrinia furnacalis* (Lepidoptera: Crambidae) eggs. Bulletin of Entomological Research, 107(6), 812–819. 28397638 10.1017/S0007485317000335

[eva13252-bib-0059] Iacovone, A., Ris, N., Poirié, M., & Gatti, J.‐L. (2018). Time‐course analysis of *Drosophila suzukii* interaction with endoparasitoid wasps evidences a delayed encapsulation response compared to *D. melanogaster* . PLoS One, 13(8). 10.1371/journal.pone.0201573 PMC6072091 30070997

[eva13252-bib-0060] Jaenike, J. (1978). On optimal oviposition behavior in phytophagous insects. Theoretical Population Biology, 14(3), 350–356. 751265 10.1016/0040-5809(78)90012-6

[eva13252-bib-0061] Jaenike, J. (1990). Host specialization in phytophagous insects. Annual Review of Ecology and Systematics, 21(1), 243–273.

[eva13252-bib-0062] Jervis, M. A., Ellers, J., & Harvey, J. A. (2008). Resource acquisition, allocation, and utilization in parasitoid reproductive strategies. Annual Review of Entomology, 53, 361–385. 10.1146/annurev.ento.53.103106.093433 17877453

[eva13252-bib-0063] Kacsoh, B. Z., & Schlenke, T. A. (2012). High hemocyte load is associated with increased resistance against parasitoids in *Drosophila suzukii*, a relative of *D. melanogaster* . PLoS One, 7(4), e34721. 22529929 10.1371/journal.pone.0034721 PMC3328493

[eva13252-bib-0064] Karageorgi, M., Bräcker, L. B., Lebreton, S., Minervino, C., Cavey, M., Siju, K. P., Grunwald Kadow, I. C., Gompel, N., & Prud'homme, B. (2017). Evolution of multiple sensory systems drives novel egg‐laying behavior in the fruit pest *Drosophila suzukii* . Current Biology, 27(6), 847–853. 28285999 10.1016/j.cub.2017.01.055 PMC5364372

[eva13252-bib-0065] Kaser, J. M., Nielsen, A. L., & Abram, P. (2018). Biological control effects of non‐reproductive host mortality caused by insect parasitoids. Ecological Applications, 28(4), 1081–1092. 29485221 10.1002/eap.1712

[eva13252-bib-0066] Keane, R. M., & Crawley, M. J. (2002). Exotic plant invasions and the enemy release hypothesis. Trends in Ecology & Evolution, 17(4), 164–170.

[eva13252-bib-0067] Keesey, I. W., Knaden, M., & Hansson, B. S. (2015). Olfactory specialization in *Drosophila suzukii* supports an ecological shift in host preference from rotten to fresh fruit. Journal of Chemical Ecology, 41(2), 121–128. 25618323 10.1007/s10886-015-0544-3 PMC4351439

[eva13252-bib-0068] Knoll, V., Ellenbroek, T., Romeis, J., & Collatz, J. (2017). Seasonal and regional presence of hymenopteran parasitoids of Drosophila in Switzerland and their ability to parasitize the invasive *Drosophila suzukii* . Scientific Reports, 7(1), 1–11. 28098183 10.1038/srep40697 PMC5241644

[eva13252-bib-0069] Kohyama, T. I., & Kimura, M. T. (2015). Toxicity of venom of *Asobara* and *Leptopilina* species to *Drosophila* species. Physiological Entomology, 40(4), 304–308.

[eva13252-bib-0070] Kraaijeveld, A., Ferrari, J., & Godfray, H. (2002). Costs of resistance in insect‐parasite and insect‐parasitoid interactions. Parasitology, 125(7), S71–S82. 12622330 10.1017/s0031182002001750

[eva13252-bib-0071] Kraaijeveld, A., Hutcheson, K. A., Limentani, E. C., & Godfray, H. C. J. (2001). Costs of counterdefenses to host resistance in a parasitoid of *Drosophila* . Evolution, 55(9), 1815–1821. 11681736 10.1111/j.0014-3820.2001.tb00830.x

[eva13252-bib-0072] Kruitwagen, A., Beukeboom, L. W., & Wertheim, B. (2018). Optimization of native biocontrol agents, with parasitoids of the invasive pest *Drosophila suzukii* as an example. Evolutionary Applications, 11(9), 1473–1497. 30344621 10.1111/eva.12648 PMC6183459

[eva13252-bib-0073] Lenth, R. (2020). emmeans: Estimated marginal means, aka least‐squares means. R package version 1.4.6. https://CRAN.R‐project.org/package=emmeans.

[eva13252-bib-0074] Lirakis, M., & Magalhães, S. (2019). Does experimental evolution produce better biological control agents? A critical review of the evidence. Entomologia Experimentalis Et Applicata, 167(7), 584–597.

[eva13252-bib-0075] Lommen, S. T., de Jong, P. W., & Pannebakker, B. A. (2017). It is time to bridge the gap between exploring and exploiting: Prospects for utilizing intraspecific genetic variation to optimize arthropods for augmentative pest control–A review. Entomologia Experimentalis Et Applicata, 162(2), 108–123.

[eva13252-bib-0076] Lynch, M., & Walsh, B. (1998). Genetics and analysis of quantitative traits (Vol. 1). Sinauer.

[eva13252-bib-0077] Maron, J. L., & Vilà, M. (2001). When do herbivores affect plant invasion? Evidence for the natural enemies and biotic resistance hypotheses. Oikos, 95(3), 361–373.

[eva13252-bib-0078] Mathe‐Hubert, H., Kremmer, L., Colinet, D., Gatti, J.‐L., Van Baaren, J., Delava, E., & Poirie, M. (2019). Variation in the venom of parasitic wasps, drift, or selection? Insights from a multivariate QST analysis. Frontiers in Ecology and Evolution, 7, 156.

[eva13252-bib-0079] Mazzetto, F., Marchetti, E., Amiresmaeili, N., Sacco, D., Francati, S., Jucker, C., Dindo, M. L., Lupi, D., & Tavella, L. (2016). *Drosophila* parasitoids in northern Italy and their potential to attack the exotic pest *Drosophila suzukii* . Journal of Pest Science, 89(3), 837–850.

[eva13252-bib-0080] Miller, B., Anfora, G., Buffington, M., Daane, K. M., Dalton, D. T., Hoelmer, K. M., Valerio, M., Stacconi, V. R., Grassi, A., Ioriatti, C., Loni, A., Miller, J. C., Ouantar, M., Wang, X., Wiman, N. G., & Walton, V. (2015). Seasonal occurrence of resident parasitoids associated with *Drosophila suzukii* in two small fruit production regions of Italy and the USA. Bulletin of Insectology, 68(2), 255–263.

[eva13252-bib-0081] Mollema, C. (1990). Heritability estimates of host selection behaviour by the *Drosophila* parasitoid *Asobara tabida* . Netherlands Journal of Zoology, 41(2–3), 174–183.

[eva13252-bib-0082] Moret, Y., & Schmid‐Hempel, P. (2000). Survival for immunity: The price of immune system activation for bumblebee workers. Science, 290(5494), 1166–1168. 11073456 10.1126/science.290.5494.1166

[eva13252-bib-0083] Münster‐Swendsen, M. (2002). Population cycles of the spruce needle miner in Denmark driven by interactions with insect parasitoids (A. Berryman (Ed.)). Oxford Univ. Press.

[eva13252-bib-0084] Paini, D. R., Sheppard, A. W., Cook, D. C., De Barro, P. J., Worner, S. P., & Thomas, M. B. (2016). Global threat to agriculture from invasive species. Proceedings of the National Academy of Sciences of the United States of America, 113(27), 7575–7579. 27325781 10.1073/pnas.1602205113 PMC4941431

[eva13252-bib-0085] Pejchar, L., & Mooney, H. A. (2009). Invasive species, ecosystem services and human well‐being. Trends in Ecology & Evolution, 24(9), 497–504. 19577817 10.1016/j.tree.2009.03.016

[eva13252-bib-0086] Phillips, B. L., & Shine, R. (2004). Adapting to an invasive species: Toxic cane toads induce morphological change in Australian snakes. Proceedings of the National Academy of Sciences of the United States of America, 101(49), 17150–17155. 15569943 10.1073/pnas.0406440101 PMC535375

[eva13252-bib-0087] Poirié, M., Carton, Y., & Dubuffet, A. (2009). Virulence strategies in parasitoid Hymenoptera as an example of adaptive diversity. Comptes Rendus Biologies, 332(2–3), 311–320. 19281961 10.1016/j.crvi.2008.09.004

[eva13252-bib-0088] Poyet, M., Havard, S., Prevost, G., Chabrerie, O., Doury, G., Gibert, P., & Eslin, P. (2013). Resistance of *Drosophila suzukii* to the larval parasitoids *Leptopilina heterotoma* and *Asobara japonica* is related to haemocyte load. Physiological Entomology, 38(1), 45–53.

[eva13252-bib-0089] R Core Team (2020). R: A language and environment for statistical computing. R Foundation for Statistical Computing.

[eva13252-bib-0090] Rauw, W. M. (2012). Immune response from a resource allocation perspective. Frontiers in Genetics, 3, 267. 23413205 10.3389/fgene.2012.00267 PMC3571735

[eva13252-bib-0091] Ris, N., Allemand, R., Fouillet, P., & Fleury, F. (2004). The joint effect of temperature and host species induce complex genotype‐by‐environment interactions in the larval parasitoid of *Drosophila*, *Leptopilina heterotoma* (Hymenoptera: Figitidae). Oikos, 106(3), 451–456.

[eva13252-bib-0092] Rizki, R., & Rizki, T. (1990). Parasitoid virus‐like particles destroy *Drosophila* cellular immunity. Proceedings of the National Academy of Sciences of the United States of America, 87(21), 8388–8392. 2122461 10.1073/pnas.87.21.8388 PMC54961

[eva13252-bib-0093] Robertson, B. A., Rehage, J. S., & Sih, A. (2013). Ecological novelty and the emergence of evolutionary traps. Trends in Ecology & Evolution, 28(9), 552–560. 23756104 10.1016/j.tree.2013.04.004

[eva13252-bib-0094] Rolff, J., & Kraaijeveld, A. (2001). Host preference and survival in selected lines of a *Drosophila* parasitoid, *Asobara tabida* . Journal of Evolutionary Biology, 14(5), 742–745.

[eva13252-bib-0095] Rosenheim, J. A., & Rosen, D. (1992). Influence of egg load and host size on host‐feeding behaviour of the parasitoid *Aphytis lingnanensis* . Ecological Entomology, 17(3), 263–272.

[eva13252-bib-0096] Rossbacher, S., & Vorburger, C. (2020). Prior adaptation of parasitoids improves biological control of symbiont‐protected pests. Evolutionary Applications, 13(8), 1868–1876. 10.1111/eva.12934 32908591 PMC7463345

[eva13252-bib-0097] Rossi Stacconi, M. V., Buffington, M., Daane, K. M., Dalton, D. T., Grassi, A., Kaçar, G., Miller, B., Miller, J. C., Baser, N., Ioriatti, C., Walton, V. M., Wiman, N. G., Wang, X., & Anfora, G. (2015). Host stage preference, efficacy and fecundity of parasitoids attacking *Drosophila suzukii* in newly invaded areas. Biological Control, 84, 28–35.

[eva13252-bib-0098] Roy, H., Handley, L.‐J.‐L., Schönrogge, K., Poland, R., & Purse, B. (2011). Can the enemy release hypothesis explain the success of invasive alien predators and parasitoids? BioControl, 56(4), 451–468.

[eva13252-bib-0099] Ruschioni, S., van Loon, J. J., Smid, H. M., & van Lenteren, J. C. (2015). Insects can count: Sensory basis of host discrimination in parasitoid wasps revealed. PLoS One, 10(10), e0138045. 26466380 10.1371/journal.pone.0138045 PMC4605743

[eva13252-bib-0100] Samson‐Boshuizen, M., Bakker, K., & van Lenteren, J. C. (1973). Success of parasitization of *Pseudeucoila bochei* Weld (Hym., Cynip.): A matter of experience. Netherlands Journal of Zoology, 24(1), 67–85.

[eva13252-bib-0101] Schlaepfer, M. A., Runge, M. C., & Sherman, P. W. (2002). Ecological and evolutionary traps. Trends in Ecology & Evolution, 17(10), 474–480.

[eva13252-bib-0102] Schlaepfer, M. A., Sherman, P. W., Blossey, B., & Runge, M. C. (2005). Introduced species as evolutionary traps. Ecology Letters, 8(3), 241–246.

[eva13252-bib-0103] Schwenke, R. A., Lazzaro, B. P., & Wolfner, M. F. (2016). Reproduction–immunity trade‐offs in insects. Annual Review of Entomology, 61, 239–256. 10.1146/annurev-ento-010715-023924 PMC5231921 26667271

[eva13252-bib-0104] Stacconi, M. V. R., Panel, A., Baser, N., Ioriatti, C., Pantezzi, T., & Anfora, G. (2017). Comparative life history traits of indigenous Italian parasitoids of *Drosophila suzukii* and their effectiveness at different temperatures. Biological Control, 112, 20–27.

[eva13252-bib-0105] Stahl, J., Tortorici, F., Pontini, M., Bon, M.‐C., Hoelmer, K., Marazzi, C., Tavella, L., & Haye, T. (2019). First discovery of adventive populations of *Trissolcus japonicus* in Europe. Journal of Pest Science, 92(2), 371–379.

[eva13252-bib-0106] Stotz, G. C., Gianoli, E., & Cahill, J. F. (2016). Spatial pattern of invasion and the evolutionary responses of native plant species. Evolutionary Applications, 9(8), 939–951. 27606003 10.1111/eva.12398 PMC4999525

[eva13252-bib-0107] Strand, M. R., & Pech, L. L. (1995). Immunological basis for compatibility in parasitoid‐host relationships. Annual Review of Entomology, 40(1), 31–56. 10.1146/annurev.en.40.010195.000335 7810989

[eva13252-bib-0108] Strauss, S. Y., Lau, J. A., & Carroll, S. P. (2006). Evolutionary responses of natives to introduced species: What do introductions tell us about natural communities? Ecology Letters, 9(3), 357–374. 16958902 10.1111/j.1461-0248.2005.00874.x

[eva13252-bib-0109] Thompson, J. N. (1988). Evolutionary ecology of the relationship between oviposition preference and performance of offspring in phytophagous insects. Entomologia Experimentalis Et Applicata, 47(1), 3–14.

[eva13252-bib-0110] van Lenteren, J. C. (2012). The state of commercial augmentative biological control: Plenty of natural enemies, but a frustrating lack of uptake. BioControl, 57(1), 1–20.

[eva13252-bib-0111] van Lenteren, J. C., Isidoro, N., & Bin, F. (1998). Functional anatomy of the ovipositor clip in the parasitoid *Leptopilina heterotoma* (Thompson) (Hymenoptera: Eucoilidae), a structure to grip escaping host larvae. International Journal of Insect Morphology and Embryology, 27(3), 263–268.

[eva13252-bib-0112] van Lenteren, J. C., Ruschioni, S., Romani, R., van Loon, J. J. A., Qiu, Y. T., Smid, H. M., Isidoro, N., & Bin, F. (2007). Structure and electrophysiological responses of gustatory organs on the ovipositor of the parasitoid *Leptopilina heterotoma* . Arthropod Structure & Development, 36(3), 271–276. 18089105 10.1016/j.asd.2007.02.001

[eva13252-bib-0113] Via, S., & Hawthorne, D. J. (2002). The genetic architecture of ecological specialization: Correlated gene effects on host use and habitat choice in pea aphids. The American Naturalist, 159(S3), S76–S88. 10.1086/338374 18707371

[eva13252-bib-0114] Vinson, S. B., & Iwantsch, G. (1980). Host suitability for insect parasitoids. Annual Review of Entomology, 25(1), 397–419.

[eva13252-bib-0115] Wajnberg, E. (2004). Measuring genetic variation in natural enemies used for biological control: Why and how. Genetics, Evolution and Biological Control, 19–37.

[eva13252-bib-0116] Weber, K., & Diggins, L. (1990). Increased selection response in larger populations. II. Selection for ethanol vapor resistance in *Drosophila melanogaster* at two population sizes. Genetics, 125(3), 585–597. 2116359 10.1093/genetics/125.3.585 PMC1204085

[eva13252-bib-0117] Wertheim, B., Kraaijeveld, A. R., Hopkins, M. G., Walther, B. M., & Godfray, H. C. J. (2011). Functional genomics of the evolution of increased resistance to parasitism in *Drosophila* . Molecular Ecology, 20(5), 932–949. 21062384 10.1111/j.1365-294X.2010.04911.x

[eva13252-bib-0118] Wilson, A. J., Réale, D., Clements, M. N., Morrissey, M. M., Postma, E., Walling, C. A., Kruuk, L. E. B., & Nussey, D. H. (2010). An ecologist's guide to the animal model. Journal of Animal Ecology, 79(1), 13–26. 10.1111/j.1365-2656.2009.01639.x 20409158

[eva13252-bib-0119] Wricke, G., & Weber, E. (1986). Quantitative genetics and selection in plant breeding. Walter de Gruyter.

